# Leveraging quality improvement initiatives to support development of decision support tools in healthcare

**DOI:** 10.1080/20476965.2025.2500285

**Published:** 2025-05-05

**Authors:** Joe Viana, Christos Vasilakis, Neophytos Stylianou

**Affiliations:** a Department of Industrial Economics and Technology Management, NTNU – Norwegian University of Science and Technology, Trondheim, Norway; b Center for Service Innovation, St. Olav’s Hospital, Trondheim, Norway; c Department of Accounting and Operations Management, BI Norwegian Business School, Oslo, Norway; d School of Management, University of Bath, Bath, UK; e NS Intelligence Solution ltd, Lefkosia, Cyprus; f Department of Resilient Society, Eratosthenes Centre of Excellence, Limassol, Cyprus

**Keywords:** Quality improvement, discrete-event simulation, health, atrial fibrillation

## Abstract

Modelling and simulation studies have been used to inform the choices and development of quality improvement (QI) initiatives in health care, for example, by helping refine the intervention to be implemented or support decisions around the management of demand and capacity. We do not know whether a modelling study can itself be informed by a QI project and what are the associated benefits and challenges. In this research, we sought to investigate the opportunities and challenges associated with an ongoing health service-led QI project in informing the development of a stochastic simulation-based decision support tool to inform decisions around the commissioning of anticoagulation services for patients with atrial fibrillation. We found that the positive synergies offered by the QI project included good access to stakeholders and envisaged end users, co-producing relevant and impactful scenarios for experimentation, as well as access to good quality individual patient level data. On the other hand, substantial effort was required to populate input parameters with values that pertain to the natural history of the disease and the effectiveness of the different treatments. Our findings indicate that, if stakeholders require modelling results to inform aspects of a QI project, upfront investment is needed to ensure timely interaction between the two studies.

## 1. Introduction

Traditionally, modelling and simulation studies have been used to inform the choices and development of quality improvement (QI) initiatives in health care (Booker et al., 2016; Zimmerman et al., 2016). For example, modelling and simulation can help define or fine-tune the type of interventions to be implemented or support decisions around the management of patient demand and care system capacity (Comas et al., 2014; Criddle & Holt, 2018; Famiglietti et al., 2017). We do not know whether the development of decision support tool can itself be informed by a QI project and what are the benefits and challenges of such an endeavour. In this paper, we seek to address this gap in knowledge through the opportunity afforded by a project that developed a stochastic simulation-based decision support tool to inform decisions around the commissioning of anticoagulation services for patients with atrial fibrillation.

Atrial fibrillation (AF), an arrhythmia characterized by chaotic electrical activity in the atria (the two upper chambers in the heart), is a public health problem affecting countries experiencing population ageing (British Heart Foundation, 2024; Lichten et al., 2015). AF not only increases the risk of stoke five-fold, but strokes suffered by this group of patients are more significant and result in more serious harm and greater mortality than strokes in other patient groups (Adderley et al., 2018). Historically, warfarin and aspirin have been the treatments of choice for AF, but recent guidance states that aspirin should not be used as a monotherapy (Alshehri, 2019; NICE National Institute for Health and Care Excellence., 2021). More recently, a new class of drugs was developed, Novel Oral Anti-Coagulants (NOACs), which aim to treat AF while avoiding some of the drawbacks of warfarin (Hicks et al., 2016; Martinez et al., 2023). However, each treatment comes with costs and benefits for both the patient and care system which need to be considered when planning the organisation of stroke prevention services at a regional or national level. Given the lack of a standardised approach in the management of AF at the time of this study, this presented a major challenge for national and regional health care planners.

Hence, the aim of the case study was to build upon an ongoing regional QI project by developing a prototype modelling software tool to help with decisions around the organisation of anti-coagulation and stroke prevention services. The specific objectives of the case study were:

- Construct a stochastic simulation model representing individual patients with AF on different treatments and their respective outcomes.
- Implement a prototype software tool to allow the execution of computer simulation experiments relevant to different local, regional, and national health care systems looking to re-organise stroke prevention services.
- Experiment with a number of illustrative scenarios that evaluate the likely impact of changes in the mixture of medication treatments on patient and system level outcomes.

A literature search at the time of initiating the modelling study revealed that although some modelling work had been reported, most of it concerned economic evaluations of different options in the management of patients with AF (Davidson et al., 2013; Limone et al., 2014; Sullivan et al., 2006; You, 2014). To the best of our knowledge, no study has been reported in the literature which takes a wider perspective in the management of patients with AF and investigates the likely impact of changes on both patient and system outcomes at a regional health economy level. Rose et al. (2011) did report on a model developed in this area; however, it was a one-off modelling study that did not result in a tool that can be used in other settings, while the population-level characteristics and health policy levers were incompatible with those of our study. Finally, there were no reported studies that had have incorporated the most recent UK National Institutes for Health and Care Excellence (NICE) guidelines on the management of patients with AF at the time (NICE National Institute for Health and Care Excellence, 2021).

Building up from the modelling case study in the management of patients with AF, we sought to answer the following overarching research question: what are the opportunities and challenges associated with a live QI project to inform the development of a stochastic simulation decision support tool? We found that the positive synergies offered by the QI project included good access to stakeholders and envisaged end users, co-producing relevant and impactful scenarios for experimentation, as well as access to individual patient level data that were used in defining the simulated patient cohort. On the other hand, additional and substantial effort was required to populate the model with values for those model input parameters that pertain to the natural history of the considered disease and the clinical effectiveness of the different treatments.

## 2. Background and literature review

### 2.1. Quality improvement

Quality improvement (QI), often defined as the use of methods and tools to continuously improve quality of care and outcomes for patients (Alderwick et al., 2017), can trace its origins and maintains strong conceptual link with many operations management principles and tools including quality management, business process management (Amaral et al., 2011), Kaizen (Mazzocato et al., 2016), PDSA cycles (O’Connor, 2020), etc. More specifically, it involves the systematic and coordinated approach to solving a problem using specific methods and tools with the aim of bringing about a measurable improvement (The Health Foundation., 2021). QI comes in many forms and aligns with the scientific experimental method (Zann et al., 2021) through the provision of data-based feedback (Zimmerman et al., 2016). The UK’s National Institute for Health and Care Excellence (NICE National Institute for Health and Care Excellence., 2022) suggests the role quality standards and QI is critical and comprehensive including in identifying gaps and areas for improvement, measuring the quality of care, understanding how to improve care, demonstrating that care organisations provide quality care and commission high-quality services.

QI projects simultaneously address the quality of care and the sustainability of the institution(s) providing that care, working to improve efficiency and maximise access and patient flow while safeguarding patient safety. QI is linked to performance improvement and can help organisations maximise the care they can deliver, and in some instances do more with less. Targeted interventions, even of minor inefficiencies, can have profound impact on patient care and the available resources (Rutberg et al., 2015). The objectives of targeted interventions which focus on a specific patient population or processes in a hospital can be easy to define but difficult to assess, due to the complexity of the system or interconnected systems involved, which is where modelling and simulation can help.

### 2.2. Modelling and simulation in healthcare improvement

A model according to Pidd (2010) “is an external and explicit representation of part of reality as seen by the people who wish to use that model to understand, to change, to manage and to control that part of reality”, which is echoed by others (Brailsford et al., 2009; Caro et al., 2012; Sterman, 2000). It is known that the different qualitative and quantitative modelling techniques can support QI (Brailsford et al., 2009; Crowe et al., 2017; Pitt et al., 2016). The portfolio of modelling and simulation techniques is vast, with the characteristics of the system being modelled and the type of decision support required determining the choice of modelling method(s) to be used to address the problem at hand (Brailsford et al., 2009; Pitt et al., 2016), but the process can be challenging (Eldabi et al., 2007; Harper & Pitt, 2004). Often the more technical methods can be combined with qualitative problem structuring methods such as Soft System Methodology (Crowe et al., 2017) and Causal Loop Diagrams (Sterman, 2000). These qualitative methods can be used to frame the problem prior to assessment of potential changes to the system (Crowe et al., 2017; Holm et al., 2013; Sachdeva et al., 2007; Tako & Kotiadis, 2015) such as Discrete Event Simulation (DES), System Dynamics (SD), Agent Based Modelling (ABM) Monte Carlo Simulation and Mathematical Modelling (Brailsford et al., 2009; Katsaliaki & Mustafee, 2011).

DES, which has been extensively used in healthcare (Brailsford et al., 2009; Fone et al., 2003; Günal & Pidd, 2010; Jacobson et al., 2013; Karnon et al., 2012; Katsaliaki & Mustafee, 2011), is a stochastic simulation modelling approach suitable for queuing systems. The extensive DES modelling use in healthcare is evident from the reviews presented, and although many of these studies address QI type problems, they are not classified or referred to in the paper as such. Rutberg et al. (2015) argues that DES is not a QI method but can support existing QI frameworks including Lean, and Six Sigma.

### 2.3. Modelling and simulation in support of QI initiatives

Traditionally, modelling and simulation studies have been used (or intended) to inform the choices and development of healthcare improvement initiatives; however, few studies clearly state they were part of a formal QI project. For example, Viana et al. (2020) used DES to investigate the operation of a post-term pregnancy outpatient clinic, the study was not a formal QI project, yet implementation of the study’s recommendations did lead to improvements in clinic performance. A Web Of Science search (Search terms: AB= (“quality improvement”) AND AB = (simulat*) AND AB = (health)) identified 174 articles. Following an initial review 17 articles were included and an additional 16 articles were identified through backward reference searching (Table 1). The literature strategy is described in more detail in Appendix C. It is important here to clarify that there is a difference between general efforts to improve quality and QI, which is a specific method (or set of methods) for improving quality as described in section 2.1. We attempted to select papers that specifically refer to QI and/or continuous improvement as a method rather than as general concept.

**Table 1.** An overview of the studies identified in the scoping literature review.

Source	Year	Care setting(s)	Place	Informed QI vs. QI informed	Findings applied?
Sturm and Wells	1995	Mental health care	USA	Informs	No
Coelli et al.	2007	Hospital & private mammography clinic	Brazil	Informs	No
Iwashyna et al.	2009	Network of hospital ICUs and EDs	USA	Informs	No
Santibáñez et al.	2009	Cancer Centre Ambulatory Care Unit	Canada	Informs	No
Booker et al.	2016	Hospital Radiology	USA	Informs	No
Pandya et al.	2020	Acute ischemic stroke care	USA	Informs	No
Spencer et al.	2020	HPV Vaccination uptake	USA	Informs	No
Zimmerman et al.	2016	Hospital Mental Health Outpatient	USA	Informs	No
Hung et al.	2007	Hospital Paediatric ED	Canada	Informs	Unclear
Ferreira et al.	2008	Hospital surgical centre	Brazil	Informs	Unclear
Nickel and Schmidt	2009	Hospital Radiology	Germany	Informs	Unclear
Corazza et al.	2011	Hospital radiation therapy centre	Switzerland	Informs	Unclear
Robinson et al.	2012	Theatres; Paediatrics; Cystic Fibrosis clinic	UK	Informs	Unclear
Comas et al.	2014	Breast cancer screening	Spain	Informs	Unclear
Carney et al.	2015	Community Health Centre Cancer screening	USA	Informs	Unclear
Famiglietti et al.	2017	Radiation Oncology treatment centre	USA	Informs	Unclear
Huang et al.	2007	Federal Community Health Centers	USA	Informs	Yes
Brenner et al.	2010	University Hospital Emergency Department (ED)	USA	Informs	Yes
Rose et al.	2011	Anticoagulation for atrial fibrillation	USA	Informs	Yes
Celano et al.	2012	Hospital ED	Italy	Informs	Yes
Paul and Lin	2012	Hospital ED	USA	Informs	Yes
Day et al.	2013	Hospital ED	USA	Informs	Yes
Marmor et al.	2013	Hospital Nuclear Medicine	USA	Informs	Yes
Day et al.	2015	Hospital Cardiac Intensive Care Unit (ICU)	USA	Informs	Yes
McKetta et al.	2016	Hospital Paediatric Procedure Complex	USA	Informs	Yes
Criddle and Holt	2018	Paediatric Hospital Post Anaesthesia Care Unit	USA	Informs	Yes
This paper	–	Anticoagulation for atrial fibrillation and stroke prevention services	UK	Informed by	Ongoing

Studies in which model recommendations have been implemented at the time of publication are uncommon but include the use of DES to improve paediatric surgery services (McKetta et al., 2016) and associated paediatric post anaesthesia care unit (Criddle & Holt, 2018). Zimmerman et al. (2016) developed an SD model to streamline posttraumatic stress disorder specialist referrals. DES has been used to improve emergency department (ED) performance, through various operational changes and reconfigurations, e.g., staff/resource availability (Brenner et al., 2010; Celano et al., 2012; Day et al., 2013; Paul & Lin, 2012). Other related models with implemented recommendations include DES models to improve Cardiac Intensive Care unit operation (Day et al., 2015) and access to positron emission tomographic scans (Marmor et al., 2013). Robinson et al. (2012) present a SimLean approach in which DES can be used to educate, facilitate, and evaluate three Lean healthcare example projects.

Many studies do not report if model recommendations were implemented e.g., Iwashyna et al. (2009) network analysis of the regionalisation of critical care services. ED related models focusing on ambulatory care (Santibáñez et al., 2009), paediatric ED (Hung et al., 2007) and a surgical centre (Ferreira et al., 2008). Similarly, cancer screening and associated treatment services are extensively studied, for reviews see (Hollingworth & Spackman, 2007; Lindsköld et al., 2008), yet many of these studies’ present recommendations only. Simulation has been used to assess cancer screening programs (Carney et al., 2014; Comas et al., 2014) and improve radiology patient flow (Coelli et al., 2007; Corazza et al., 2011; Famiglietti et al., 2017; Ju et al., 2015; Nickel & Schmidt, 2009). Carney et al. (2015) present a computational modelling approach combining health services research, health informatics, network theory and system science principles to evaluate cancer screening QI efforts.

Earlier work, by Sturm and Wells (1995) used simulation to investigate cost effectiveness of QI measures to improve depression care. The model simulated the effects of different processes within different care specialities (e.g., general medicine, psychiatry). There are many examples of modelling and simulation used to support economic evaluation of QI initiatives (Colbourn et al., 2015; Huang et al., 2007; Pandya et al., 2020; Shao et al., 2020; Spencer et al., 2020). The study by Rose et al. (2011), which we have already referred to in the Introduction, developed a mathematical model and used Monte Carlo simulation to demonstrate the potential for cost savings from improved control in patients anticoagulated for atrial fibrillation.

All the studies presented informed but were not informed by QI projects. Hence, we sought to investigate the opportunities and challenges afforded by an ongoing QI project in developing a simulation-based decision support tool to inform decisions around the commissioning of anticoagulation services for patients with atrial fibrillation. The long-term vision is for such tools, developed in parallel with pioneering QI projects (such as the one informing the case study reported here), to assist in spreading and scaling up innovation and improvement in different care settings – a known major challenge (Greenhalgh & Papoutsi, 2019).

## 3. Tool development

### 3.1. Modelling methodologies

The model could be developed using several approaches including analytical modelling, system dynamics (SD) and discrete event simulation (DES).

With analytical modelling we could only capture a few of the essential components of the whole system and the flows of patients between those components. Analytical modelling would not allow for much detail of the organisation of the care system to be included in a realistic way. For example, it would be difficult to include many (or any) stochastic elements such as random effects and uncertainties especially at the individual-patient level (e.g., time between onset of AF and stroke). Nevertheless, such an approach could potentially be a steppingstone (e.g., scoping the problem, identifying data needs) and the resulting model would also be easier to implement into a computerised software tool if required.

With SD modelling aggregate flows of patients, any feedback effect in the care system (which occurs when outputs of part of the system are “fed back” as inputs to another part) and the effects of time delays and non-linear relationships between these flows can potentially be captured. Again, the SD methodology does not allow for random effects or information at individual-patient level to be captured. However, it would allow for the analysis of the dynamic interactions between the system components and variables and how these “play out” over time.

DES is a technique whereby real-life systems are simulated. In this technique all the processes in a complex system are coded, and the series of events that occur over time are modelled. With DES individual patients and their unique trajectories as they flow through the entire care system could be captured. Random effects and many different patient attributes such as age, gender, *CHA*DS_2_ score (a risk stratification score, Gage et al., 2001) etc. can be included in the modelling. The models can run over extended time horizons where patients move through the modelled system as they experience events at discrete points in simulated time. DES can provide the flexibility to incorporate capacity and resource constraints explicitly and to capture the “competition” between modelled entities (e.g., patients) for access to limited resources (e.g., appointments in clinic).

DES also has drawbacks such as the need for more and finer grained data for estimating the values of input parameters, longer model implementation times and increased computational costs associated with running experiments; however, due to the nature of the research project and the specific requirements of this tool, DES was the modelling approach of choice.

### 3.2. Setting and data sources

The research was carried out as part of a QI project aimed at reducing the risk of stroke in people with AF and support an increase in the uptake of anticoagulation including new technology (NOACs) in line with NICE evidence across the region and the country at a later stage. It also aimed at optimising the use of anticoagulation and to see a decrease in the use of aspirin for stroke prevention in primary care. It was a complex programme of interventions set within primary care and was implemented in different phases each with a specific target group at a different location. The QI project was led by one of the 15 Health Innovation Networks, which were established in 2013 to spread innovation in England at pace and at scale (NHS England, 2023). HIEW The regional Network that led the QI project covers an area in England with a population of around 2.7 million people.

As with many studies, the data were “routinely” collected and as a result, not entirely fit for the purposes of this research project. That said, the dataset was very rich in respect to the epidemiological aspects of the project which proved useful in describing the input population for our models. Given that the dataset was not longitudinal in nature, it lacked several data items that were needed by the simulation model.

The data include records of 10,300 patients. Of these, 57.68% are males and 42.32% are females (see also Figure A-3 in Appendix). This is in line with what is already known about AF, i.e., it is most prevalent in the male population. Age ranged between 0–103. Mean (standard deviation) age is 75.60 (11.89) and Median (IQR) 77 (15). Within the dataset provided, the different variables that makeup the CHADS_2_ score were present: Congestive Heart Failure, Diabetes, *Age* ≥75, Hypertension, History of Stroke/TIA, from which we calculated the score (Table A-2).

In terms of input parameter values for the modelling tool and illustrative study, we used a combination of data estimates from the QI project, which we had to supplement by estimates published in the literature. The latter step, although time consuming, was necessary especially in terms of populating those parameters of the tool pertaining to the natural history of the disease and the relative effectiveness of the pharmaceutical treatments. Complete information about the input data and parameter values used in the tool can be found in Appendix A.

### 3.3. Overview of the simulation model

**Figure 1.**
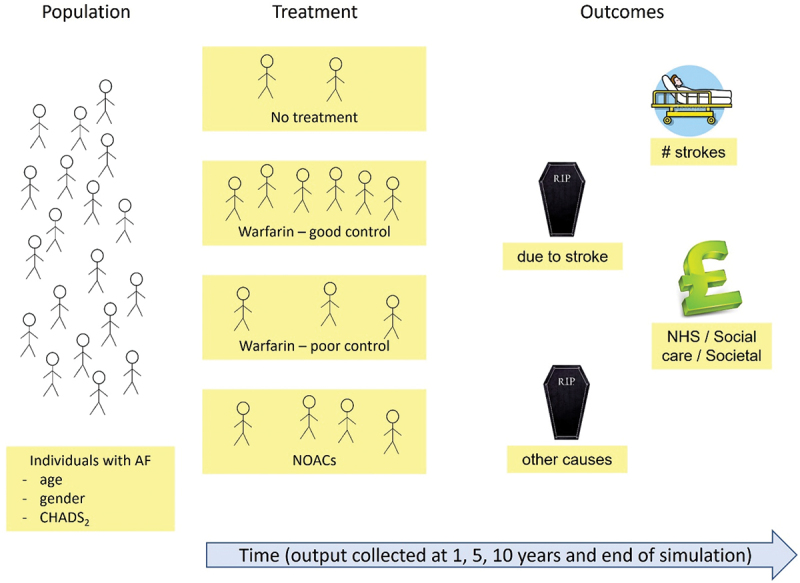
Schematic representation of the DES model. AF: atrial Fibrillation; DALY: disability adjusted life years.

The overall design of the stochastic simulation model is shown on the schematic diagram in Figure 1. Initially a population of individuals with AF is generated based on the characteristics found in the data. Treatments are allocated to these individuals based upon the rates identified in the input data. The simulated individuals are experiencing different events as time passes. Some experience stroke (ischaemic or haemorrhagic), some die because of that stroke, others die of other causes, etc. The simulation model runs until everybody which was generated in the original population dies. Data are extracted after 1, 5, and 10 years in the simulation run. Output data are the number of events of strokes, deaths from stroke, costs associated with acute care treatment, costs associated with AF treatment costs and costs associated with stroke death. Disability adjusted life years (DALYs) is also calculated. The adverse event of bleeding was included in the model design, both in terms of the risk factor (HAS-BLED score) and the outcome variable but not in the experiments due to the lack of sufficient input data.

### 3.4. Tool verification and validation

Verification and validation of the tool including the Excel user interface and the simulation model were crucial to ensure that the model was implemented correctly and to increase stakeholder confidence in the model. Key verification and validation are provided in Appendix D. Data provided by the QI project, see section 3.2 and Appendix A, were input into the simulation model via the Excel User interface. Appendix D provides face validation tests of the simulation results compared with expected results, to ensure the model correctly used this input data. Structured walk-throughs were conducted with stakeholders during several meetings, where the model as a whole and components were validated. The stakeholders confirmed that the conceptual model illustrated in Figure 1 was appropriately represented in the simulation model.

### 3.5. Tool implementation

The simulation tool was developed using the Simul8 software package (https://www.simul8.com/) and MS Excel for user interface purposes. For full documentation see Appendix B, based on the STRESS guidelines (Monks et al., 2018). The model accepts user defined input data and using probability distributions, it generates a population of a certain size with specific characteristics such as gender, age distributions, history of stroke, hypertension, and treatment for AF. The data input as described in the previous section generates a representation of the current situation, referred to as the baseline scenario. The end user also has the capacity to modify the input data in an additional worksheet to generate an alternative scenario (for comparison and what-if analysis purposes).

After the end of the simulation (when all patients generated at the start of each run come to the end of their “life”) the data generated are stored in an output spreadsheet. These include number of strokes, differentiation between haemorrhagic and ischaemic strokes, number of deaths and the costs associated with those. Outputs are collected after 1, 5, 10 years and when the simulation ends. The outputs are generated for both baseline scenario, alternative and a statistically sound comparison of the two sets of output is provided. More information about the user interface and the simulation model is presented in Appendix A and Appendix B.

## 4. Illustrative experimentation with the tool

### 4.1. Simulation parameters

The time step of the model was set to one year. This means that all system events (e.g., a simulated patient experiencing a stroke) occur at a yearly internal. The choice was dictated by both the nature of input parameters (e.g., survival rates being reported in terms of years) and computational efficiency (the finer grained the time step, the more computations required by the simulation).

Each simulation iteration (run) starts from “emptiness” and ends when the last simulated patient dies (of all causes or a stroke). Before the simulation clock starts ticking, the simulated (artificial) patient population is generated using the characteristics derived from the QI project data. Each simulated patient has the following attributes:

- Age
- Gender
- CHADS_2_ score

**Figure 2.**
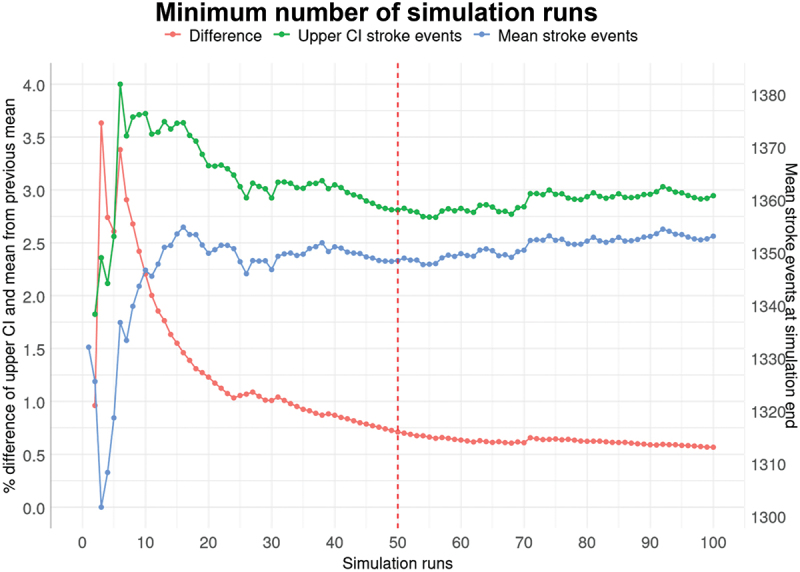
Minimum number of iterations calculation (CI: confidence interval).

As the model is stochastic, we explored the minimum number of iterations by identifying the difference between the upper confidence interval of every run and the previous runs mean. When that difference is less than 1% it is generally accepted that iterations from that point forward are not needed and are “wasting” computational resources (Hoad et al., 2010). In order to test the minimum iteration number, we used the same randomisation for the model to start with and plotted the difference in upper bound confidence interval and the mean difference from the previous run. The results are shown in Figure 2. We opted for 50 iterations as this way we were below the acceptable 1% difference, and the computational time was not significantly different.

### 4.2. Sensitivity analysis

The robustness of the model was assessed using sensitivity analysis, which allows modellers, stakeholders, and end users to increase the level of confidence in the model so that any results will be more trustworthy (Kunc et al., 2018; Pidd, 2010; Sterman, 2000).

We used one-way sensitivity analysis which allows the modellers to assess the impact that changes in a certain parameter will have on the model’s outcomes (results). We performed sensitivity analysis on the treatment costs and DALYs by altering the cost of NOACs and disability weight form haemorrhagic stroke incrementally. Both analyses did not reveal an unexpected change in model outputs as these were in line with the changes in the values of input parameters (both in direction and magnitude).

**Figure 3.**
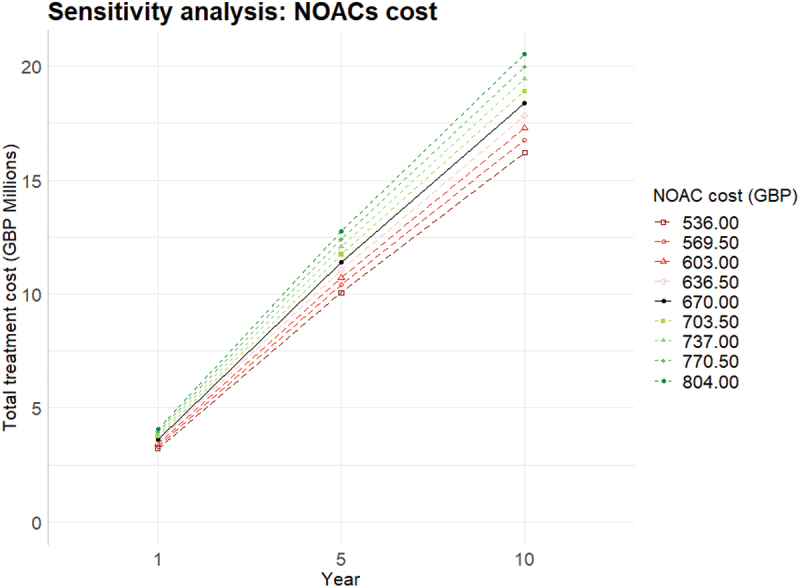
One way sensitivity analysis on costs by incrementally changing original costs of NOACs (£670).

NOACs costs was input in the model as using a triangular distribution with a peak value of £670 and max/min values of ± 10%. This was changed in increments of 5% up and down until the ± 20%. The results on the total costs of treatments for years 1, 5, and 10 are shown in Figure 3. As expected, increasing or decreasing the treatment cost and keeping all other parameters unchanged, such as number of people treated with that therapy regime, mean total treatment costs increased and decreased accordingly around the baseline figure. The output of sensitivity analysis on DALYs is reported in the Appendix (Section 4).

### 4.3. Scenarios

#### 4.3.1. Baseline scenario

**Figure 4.**
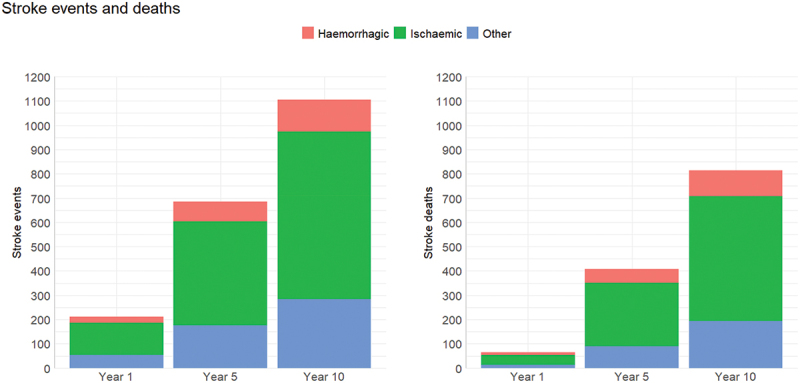
Baseline scenario: mean number of stroke events and deaths.

**Figure 5.**
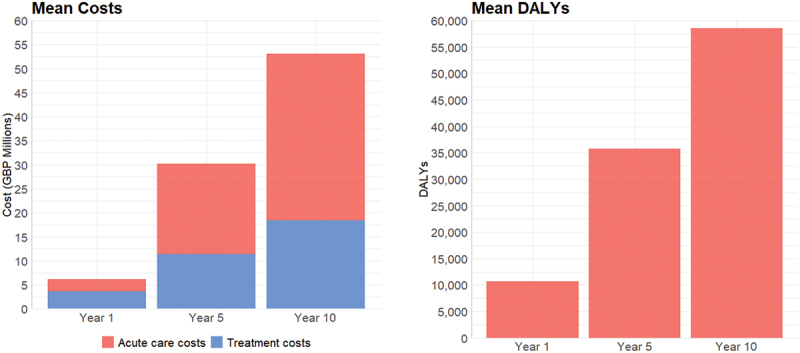
Baseline scenario: mean stroke costs and DALYs.

The model was set to run the input data without any changes to observe how the current situation would evolve over the years if the basic conditions remained unchanged. We refer to this as the baseline scenario. As expected, all outcomes showed the same gradual increase as simulation time was passing. Stroke events were increasing as well as deaths from strokes (Figure 4). This increase was to be expected given the cumulative nature of these values. The same was true for the costs and DALYs (Figure 5). Costs also increased as years pass since treatment costs and acute care costs (including negligible costs associated with death in hospital following a stroke) are accumulated over the years (Figure 5). It should be noted that confidence intervals are calculated by the tool but were not shown in the graphs of this report as they are very narrow in all output variables, thus not informative.

After consultation with the project stakeholders a number of scenarios were articulated and explored via simulation experimentation. In this paper, we present results from one such scenario.

#### 4.3.2. Intervention: treat all those who can be treated

This scenario explored the possibility of shifting patients who should be treated according to the NICE guidelines from their no treatment status to only warfarin (Scenario 1), only NOACs (2) and a combination of warfarin and NOACs based on the proportion of warfarin and NOAC patients for each CHADS_2_ score (3) (Table 2).

**Table 2.** Simulation experimentation (NT: No treatment; NOAC: novel oral anti-Coagulants; NICE).

Scenario	S0 - Baseline			S1 - Warfarin			S2 - NOAC			S3 – NICE guidelines		
CHADS_2_	NT	War	NOAC	NT	War	NOAC	NT	War	NOAC	NT	War	NOAC
**0**	904	322	360	904	322	360	904	322	360	904	322	360
	0.57	0.20	0.23	0.57	0.20	0.23	0.57	0.20	0.23	0.57	0.20	0.23
**1**	747	912	726	747	912	726	747	912	726	747	912	726
	0.31	0.38	0.30	0.31	0.38	0.30	0.31	0.38	0.30	0.31	0.38	0.30
**2**	693	1174	894	64	1803	894	64	1174	1523	64	1531	1166
	0.25	0.43	0.32	0.02	0.65	0.32	0.02	0.43	0.55	0.02	0.55	0.42
**3**	402	760	582	37	1125	582	37	760	947	37	967	740
	0.23	0.44	0.33	0.02	0.65	0.33	0.02	0.44	0.54	0.02	0.55	0.42
**4**	233	503	437	21	715	437	21	503	649	21	616	535
	0.20	0.43	0.37	0.02	0.61	0.37	0.02	0.43	0.55	0.02	0.53	0.46
**5**	107	190	178	10	287	178	10	190	275	10	240	225
	0.23	0.40	0.37	0.02	0.60	0.37	0.02	0.40	0.58	0.02	0.51	0.47
**6**	17	29	25	1	45	25	1	29	41	1	38	32
	0.24	0.41	0.35	0.01	0.63	0.35	0.01	0.41	0.58	0.01	0.54	0.45
**Total**	**3103**	**3890**	**3202**	**1784**	**5209**	**3202**	**1784**	**3890**	**4521**	**1784**	**4626**	**3784**
	**0.30**	**0.38**	**0.31**	**0.17**	**0.51**	**0.31**	**0.17**	**0.38**	**0.44**	**0.18**	**0.45**	**0.37**

**Figure 6.**
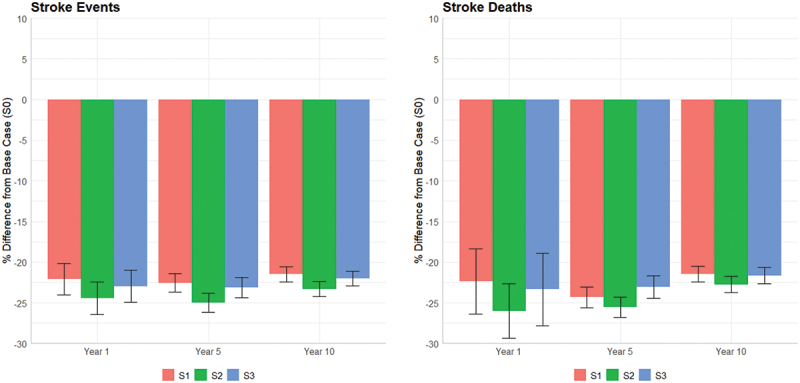
Simulation experimentation: mean absolute difference of stroke events and deaths compared to the baseline scenario.

**Figure 7.**
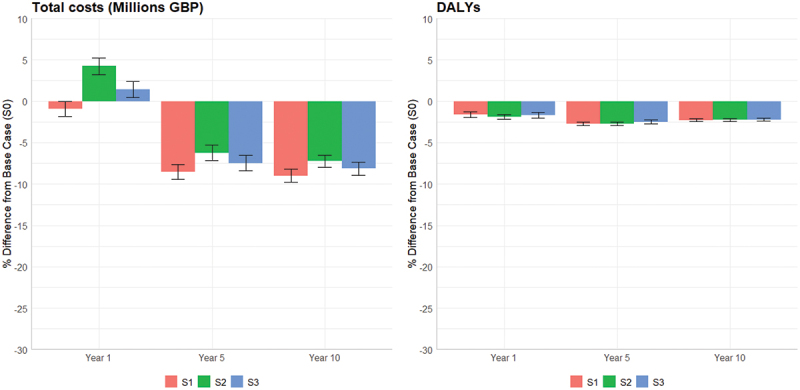
Simulation experimentation: mean absolute difference total stroke costs and DALYs compared to base case.

As expected, receiving medication resulted in considerably fewer strokes and deaths (Figure 6) compared to the baseline scenario. According to the simulation results, not only patient outcomes are likely to be improved, but total costs will also likely decrease as the increase in medication costs will be more than offset than the decrease in care cost for those patients who suffer a stroke (Figure 7). In terms of the likely impact of the scenarios on expected DALYs we observed little difference compared with the baseline model as the years of life lost in the latter case were offset by those living longer with a reduced quality of life following a stroke.

## 5. Discussion

In this paper we explored the opportunity afforded by an ongoing QI initiative in a regional health and care system to inform the development of a detailed computer simulation model and accompanying simulation study and software tool. The QI initiative sought to reduce the risk of stroke in people with AF and support an increase in the uptake of appropriate anticoagulation medications as recommended by national evidence-based guideline. We built a discrete-event individual patient simulation model in close collaboration with healthcare stakeholders, and an accompanying spreadsheet-based interface to facilitate the input and communication of the complex set of input parameter values that govern the makeup of the patient population, the risk of adverse events occurring and the relative effectiveness of the different medications available. We then carried out illustrative simulation experiments to test the likely impact of policy changes on patient and system outcomes. Although, and as mentioned in the Introduction and background sections, many studies reported in the literature have modelled the cost-effectiveness and other impacts of different options for managing the care of patients with AF, only one had done so in the context of a QI programme and their model was not directly applicable to the requirements of our study, and did not provide an evaluation of the pros and cons of doing so (Rose et al., 2011).

We found that the positive synergies offered by the QI project included good access to stakeholders and envisaged end users, co-producing relevant and impactful scenarios for experimentation, as well as access to data used to define the simulated patient cohort. Access to stakeholders and end-users was facilitated and enhanced by the modelling study running on the coattails of the main QI initiative, which helped introduce the modelling team to many stakeholders (healthcare professionals, managers, etc.) and to present interim modelling results as part of wider project meetings. Extensive interaction with these stakeholders was key in allowing for a number of simulation study components to be established including model scope and structure, modelling objectives and experimentation, establishing face validation and results interpretation. In similar fashion, the patient cohort data utilised in the model were collected and analysed by the QI project team without the need for additional primary data collection or time-consuming extractions from routine databases.

On the other hand, additional and substantial effort was required to populate the model with values for those model input parameters that pertain to the natural history of the considered disease and the clinical effectiveness of the different treatments. This task required significant investment in time and effort in searching the literature as well as collating and converting the results of the identified studies into parameter values and rates that are in the appropriate format for the model. Given the relative short time window for patient-level data collection, it was not possible to make meaningful attempts to calibrate or validate the model against regional stroke events.

An additional risk to the conduct of any applied simulation study that aims to inform decision making arises from potential delays or other problems that may be faced by the QI project, including challenges in implementation, funding, or key QI project leaders moving on. The latter is something that was experienced in this particular simulation study, with the key project leader and champion of using modelling and simulation as part of the wider initiative leaving the project before completion. Although our preliminary simulation results were presented to project stakeholders in a number of in-person meetings, by the time full simulation insights were available, project leaders were less knowledgeable of simulation modelling more generally and partly as a result, less confident in its results. This is an observation also made in a recent simulation study (Penn & Viana, 2024) and may partly explain why only a proportion of simulation studies reported in the literature demonstrate actual practical impact (Crowe et al., 2024; Sobolev et al., 2011).

As with any study, ours had a number of limitations. As this was a single case study, the ability to apply conclusions broadly across different contexts or patient populations is limited. The modelling exercise itself comes with all the limitations such studies have; models are a simplification of real-life situations without the capacity to capture all aspects of real life. Since we do not capture any other systemic effect AF may have, other than stroke events, deaths, and costs these are the only metrics we can report on. There are additional relevant healthcare as well as patient demographic and behavioural factors that were not included in the simulation study, including more detailed cost information and patient adherence to medication. Future modelling studies could capture these. Any additional future development and refinement of this or similar simulation models should consider using Free and Open Source Software platform in aid of tools that are open and shareable by design (Monks et al., 2024).

In conclusion, our insights indicate that if stakeholders require modelling results to inform aspects of a QI project, upfront investment is needed to ensure timely interaction between the two studies. Developing a simulation model (tool) as part of a QI initiative will improve chances of the accompanying innovation being taken up in other health care settings thus supporting the challenge of spreading and scaling innovation and improvement in health and care.

## Nomenclature

**Abbreviation**: **Name**
A&E/AED: Accident and Emergency Department
ABM: Agent based model
AF: Atrial fibrillation
CCG: Clinical Commissioning Group
CHADS_2_: Congestive heart failure, Hypertension, Age *>*75, Diabetes, Stroke/transient ischemic attack
CHF: Congestive heart failure
CI: Confidence Interval
DALYs: Disability adjusted life years
DES: Discrete event simulation
ED: Emergency Department
HPV: Human papillomavirus
ICU: Intensive Care Unit
IQR: Interquartile range
MCS: Monte Carlo Simulation
MS: Microsoft
NHS: The National Health Service
NICE: The National Institute for Health and Care Excellence
NOACs: Novel Oral Anti-Coagulants
ONS: The Office for National Statistics
OR: Operational Research
PACU: Post anaesthesia care unit
PDSA: Plan-do-study-act
PTSD: Posttraumatic stress disorder
QI: Quality Improvement
SD: System Dynamics
TIA: Transient ischemic attack
UK: The United Kingdom

## Supplementary Material

Appendix D Verification and Validation.docx

Appendix C PRISMA literature review.docx

Appendix B STRESS documentation.docx

Appendix A data.docx

## Data Availability

Parts of the data, such as those retrieved from the literature that support the findings of this study are available from the corresponding author, CV, upon reasonable request.
