# Supplementary material for: Leveraging quality improvement initiatives to support development of decision support tools in healthcare
Source: Health Syst (Basingstoke). 2025 May 5;14(4):323–36. doi: 10.1080/20476965.2025.2500285 (PMC12777901; doi:10.1080/20476965.2025.2500285)
Supplement: Appendix D Verification and Validation.docx [file THSS_A_2500285_SM4849.docx]

**Simulation model verification and validation**

**Verification** – Building the model correctly: compare conceptual and simulation models

**Validation** – Building correct model: compare model and real system

Validation is not an either/or proposition, *no model is ever truly valid*. Each model iteration involves some cost, time, and effort. The modeller must weigh the possible, but not guaranteed, increase in model accuracy versus the cost of increased validation effort (Banks 2014, p394).

The *purpose, or objectives, of a model must be known before it can be validated* (Robinson, 2014). This purpose may have been determined at the start of the simulation study, being expressed through the objectives.

Law (2014) defines an assumption document, as useful to document all model concepts, assumptions, algorithms, and data summaries to improve validation, and enhance the credibility of the model. An assumptions document is also known as a conceptual model (Law 2014). This is similar to the STRESS guidelines (Monks et al 2018) and project specification (Robinson 2014).

The second column of the Table on page 2 outlines key verification and validation techniques from six referenced textbooks. The third column indicates whether these techniques were used and provides references to sections of the paper, presentation, or existing model documentation where their application is described.

Following the Table on page 2 which summarises the verification and validation

**References**

1. Banks, J. (2014). **Discrete-event system simulation** (5th ed., p. 640). Pearson.
2. Hillier, F. S., & Lieberman, G. J. (2021). **Introduction to operations research** (Eleventh edition, International student edition.). McGraw-Hill Education.
3. Law, A. M. (2015). **Simulation modeling and analysis** (5th ed., p. XVIII, 776). McGraw-Hill Education.
4. Pidd, M. (2004). **Computer simulation in management science** (5th ed., p. XVI, 311). Wiley.
5. Robinson, S. (2014). **Simulation: the practice of model development and use** (2nd ed.). Palgrave Macmillan.
6. Sterman, J. D. (2000). **Business dynamics : systems thinking and modeling for a complex world** (p. XXVI, 982). Irwin McGraw-Hill.
7. Monks, T., Currie, C. S. M., Onggo, B. S., Robinson, S., Kunc, M., & Taylor, S. J. E. (2018). Strengthening the reporting of empirical simulation studies: Introducing the STRESS guidelines. *Journal of Simulation*, *13*(1), 55-67. <https://doi.org/10.1080/17477778.2018.1442155>

| **V&V** | **Validation/Verification (References)** | **Notes and references to existing model documentation and paper sections** |
| --- | --- | --- |
| *Verification building the model correctly* | 1. **Compare** **conceptual** and **simulation** models ^1, 3, 4, 5, 6^ | Yes. Stakeholders confirmed that Figure 1 in the paper and the flow chart in STRESS (Appendix B) section 2.1 were modelled correctly. |
|  | 2. Are **input parameters** represented **correctly**? ^1, 3, 4, 5, 6^ | Yes. Authors 1 and 2. Checked that the input data were used in the model. Also see the remainder of this document. |
|  | 3. Is **logical model structure** represented **correctly**? ^1, 3, 4, 5, 6^ | Yes. See 1 above. Authors discussed model specific functions with stakeholders. |
|  | 4. Engage people familiar with the system. **Experts. Structured walk through** ^1, 2, 3, 4, 5, 6^ | Yes. See 1-3 above. |
|  | 5. Get **another simulation expert to check** the simulation model. ^1, 4, 5, 6^ | Yes. Author 3, an experienced modeller, checked Author 1 and 2’s model. |
|  | 6. Create **flow diagram** which includes logical system actions & follows model logic. ^1, 3, 4, 5, 6^ | Yes. See 1 above. |
|  | 7. Examine **model output for reasonableness** under a variety of input settings. ^1, 2, 3, 4, 5, 6^ | Yes. See experimentation in the paper, sections 4.1, 4.2, and 4.3. Additional model runs were conducted for verification/validation purposes (this document) |
|  | 8. If **animation** is used verify the model logic with the animation. If graphs are considered* ^1, 2, 3, 4, 5, 6^ | Yes. Basic animation is used to track the population over time. |
|  | 9. **Debugging** (interactive run controller) step through model, **trace** individual entities. ^1, 2, 3, 5, 6^ | Yes. Patients tracked through system with set parameters to validate routing |
|  | 10. **Graphical interface** and **documentation** for verification and validation ^1, 2, 3, 4, 5, 6^ | Yes. Graphical, tabular and animation are used. |
| *Validation building correct model*  *Calibration: iterative model vs. real system comparison* | 11. Attempts to **confirm a model** is an **accurate representation** of the **real system**. ^1, 2, 3, 4, 5, 6^ | Yes. Stakeholder discussions and comparison of model results with expectations. |
|  | 12. **Model calibration**. Use **difference between model** and the real **system**, to improve the model. ^1, 3, 5, 6^ | Yes. For each mode iteration (version) input parameters were evaluated. |
|  | 13. Subjective tests: system **stakeholders, judge** the model & its output(s) & judge if model is accurate enough. Use **SSM** and **DOE**. **Turing Test(s)**, Structural assumptions. **Face validity** ^1, 2, 3, 4, 5, 6^ | Yes. Subjective comparison of service times, patient distributions, arrival patterns. |
|  | 14, Objective tests. **Statistical test(s) compare an aspect(s) of the system with the model** output data. If **purpose** changes, revalidate in terms of relevant response(s). ^1, 2, 3, 4, 5, 6^ | Not tested. |
|  | 15. **Iterative** process. **Comparing model/system** and revising the **conceptual/operational models** to accommodate perceived model deficiencies until model deemed accurate. ^1, 3, 4, 5, 6^ | Yes. The model was developed in an iterative manner. Eight versions of the model were created. |
|  | 16. **Involve model users** in model construction (conceptualization🡪implementation), to build an adequate degree of realism into the model (reasonable **assumptions** & **data**). ^1, 3, 4, 5, 6^ | Yes. Stakeholders were involved in conceptualisation, provided estimations for data distributions, and in the verification and validation of the model. |
|  | 17. **Sensitivity analysis** (check face validity). Model user asked if it **behaves in the expected way when an input variable(s) is changed**. **Extreme value tests?** ^1, 2, 3, 4, 5, 6^ | Yes. Sensitivity analysis was undertaken see paper section 4.2 and Appendix A. |
|  | 18. **Data** assumptions: based on the collection of reliable data. Data reliability: **statistical tests for homogeneity of data**. Goodness-of-fit test(s): chi-square, Kolmogorov–Smirnov test, t-Tests, distribution-free tests, *bootstrapping*, graphical methods, **confidence intervals,** % difference, correlation coefficient, *regression analysis*, Kruskal-Wallis test of homogeneity of populations, Mann-Whitney, *time series methods (spectral-analysis), correlated inspection approach*, Welch, P-P plots ^1, 3, 4, 5, 6^ | No. System stakeholders and the literature were used to determine distributions and model input values; therefore, the model data were not statistically evaluated. |
|  | 19. Can the model **predict future behaviour** of the system (inputs: data=real). Model should be accurate enough to make good predictions, for a range of input data sets. ^1, 3, 4, 5, 6^ | Yes. The recommendations from the model have not been compared against reality, as stakeholders and modellers have moved on. |
|  | 21. **Input-Output Validation**: Use **Historical Input Data**, alternative to generating input data, to drive the model and compare model output with system data. *Black box* ^1, 3, 4, 5, 6^ | Yes. In terms of population characteristics, mortality risk etc. See the remainder of this document. |
|  | 22. Validate against **another** (simulation/analytical) **model** ^2, 3, 5, 6^ | Not tested. |
|  | 23. Subjective tests: **Modeller experience** and intuition about complex systems. ^3, 5, 6^ | Yes. Author 3 (see 5 above), complimented other Authors subjective tests. |

The model generated data from 50 simulation runs is validated against the data input into the model via the Excel User Interface. Recall that this data was derived from the QI study or from the literature please see Appendix A. As some data relate to initialisation of the model, the year 1 data is used to validate that component. The data used to generate, and the associated parts of the Excel User Interface are indicated accordingly.

# Gender

**(Input-2, O16:P17; Model results from 50 runs, year 1)**

The model assignment of gender is valid as indicated in Table 1.

Table 1: Gender validation: Mean results from 50 simulation runs and 95% confidence intervals (CI)

| **Input** | **Input (%)** | **Model (%)** | **Model CI** |
| --- | --- | --- | --- |
| Male | 57.61 | 57.45 | 57.44-57.73 |
| Female | 42.39 | 42.41 | 42.27-42.55 |

# Age Bands

**(Input-2, O20:R32; Model results from 50 simulation runs, year 1)**

The model assigned ages, are valid when compared against the age band distribution data provided available and used in the input sheet as illustrated in Table 2.

Table 2: Age band validation: Mean results from 50 runs and 95% confidence intervals (CI)

| **Lower age** | **Upper age** | **Input**  **Male (%)** | **Model**  **Male (%)** | **Model CI** | **Input**  **Female (%)** | **Model**  **Female (%)** | **Model CI** |
| --- | --- | --- | --- | --- | --- | --- | --- |
| 40 | 44 | 1.06 | 1.06 | 1.02-1.10 | 0.46 | 0.46 | 0.44-0.48 |
| 45 | 49 | 1.87 | 1.89 | 1.84-1.95 | 0.97 | 0.97 | 0.93-1.00 |
| 50 | 54 | 2.84 | 2.88 | 2.82- 2.94 | 2.08 | 2.02 | 1.96-2.08 |
| 55 | 59 | 4.92 | 4.90 | 4.81-4.98 | 2.13 | 2.13 | 2.05-2.21 |
| 60 | 64 | 7.19 | 7.17 | 7.07-7.26 | 3.75 | 3.75 | 3.66-3.84 |
| 65 | 69 | 13.15 | 13.04 | 12.91-13.16 | 8.19 | 8.18 | 8.06-8.30 |
| 70 | 74 | 16.28 | 16.34 | 16.21-16.47 | 12.40 | 12.54 | 12.40-12.69 |
| 75 | 79 | 18.78 | 18.81 | 18.65-18.98 | 17.05 | 17.06 | 16.91-17.24 |
| 80 | 84 | 16.47 | 16.48 | 16.38-16.59 | 20.08 | 19.98 | 19.79-20.16 |
| 85 | 89 | 12.23 | 12.21 | 12.10-12.32 | 18.77 | 18.75 | 18.58-18.92 |
| 90 | 94 | 4.36 | 4.35 | 4.26-4.43 | 10.83 | 10.91 | 10.80-11.03 |
| 95 | 100 | 0.87 | 0.88 | 0.84-0.91 | 3.29 | 3.24 | 3.17-3.32 |

# Treatment Cost

## NOAC – Treatment costs

**(Input-3, E76:F78; Model results from 50 simulation runs, all years)**

NOAC treatment costs were based on the clinical guidelines at the time to be £675. To incorporate variability triangular distributions with a minimum and maximum of ±10% of the mean, see Figure 1, for the treatment costs for all model generated patients, plotted against a bootstrapped sample of the distribution with the given parameters.


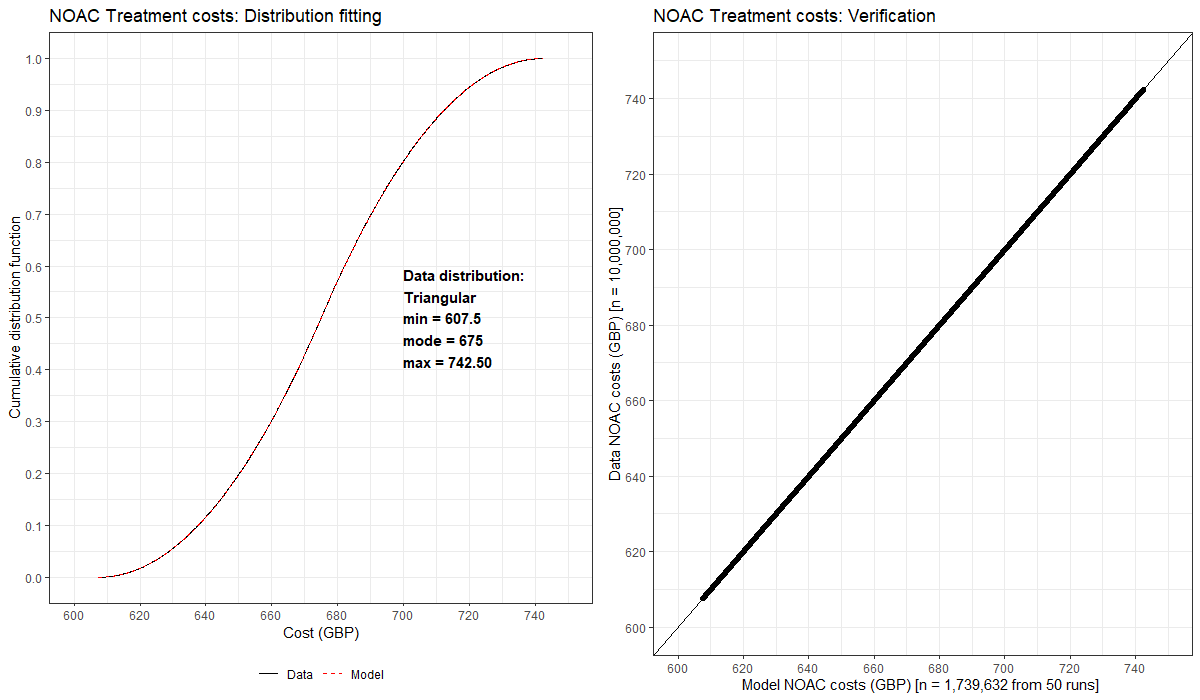


Figure 1: NOAC treatment cost validation: Distribution fitting. Results from 50 simulation runs compared to large sample

## Warfarin

**(Input-3, E76:F78; Model results from 50 runs, all years)**

Warfarin treatment costs were based on the clinical guidelines at the time to be £380. To incorporate variability triangular distributions with a minimum and maximum of ± 10% of the mean, see Figure 2, for the treatment costs for all model generated patients, plotted against a bootstrapped sample of the distribution with the given parameters.

**
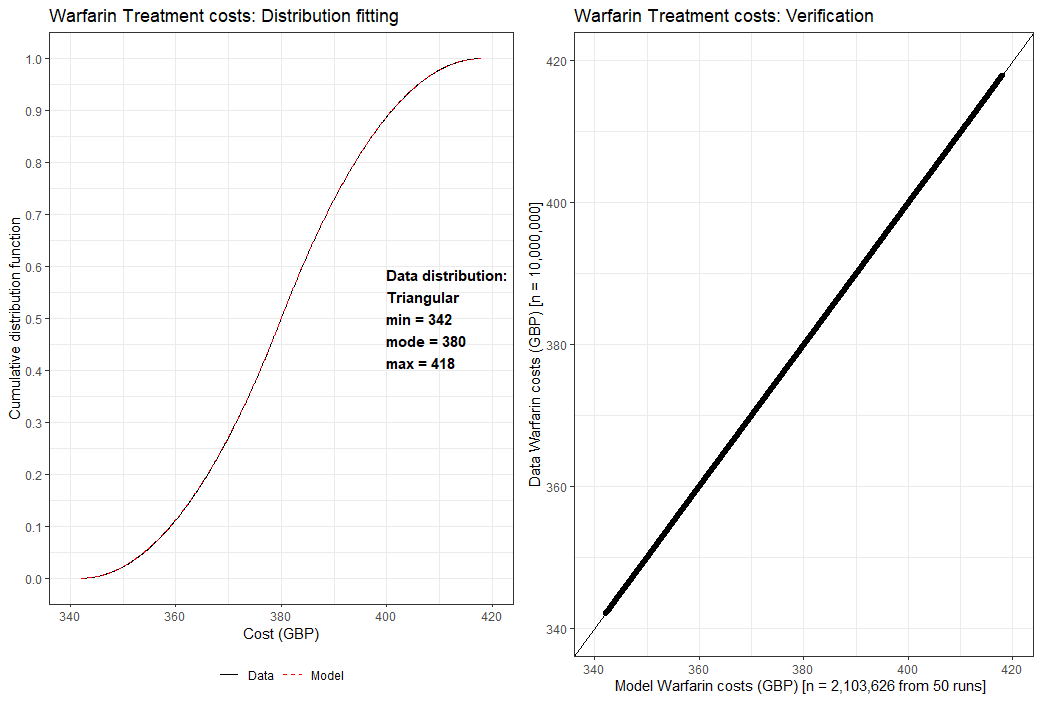
**

Figure 2: Warfarin treatment cost validation: Distribution fitting. Results from 50 simulation runs compared to large sample

# Acute Care Cost

## Year 1

**(Input-3, B76:C82; Model results from 50 simulation runs, all years)**

The first-year acute care costs at the time given by the data at the time and discussion with stakeholders was £11,900. To incorporate variability triangular distributions with a minimum and maximum of ± 10% of the mean, see Figure 3, for the treatment costs for all model generated patients, plotted against a bootstrapped sample of the distribution with the given parameters.


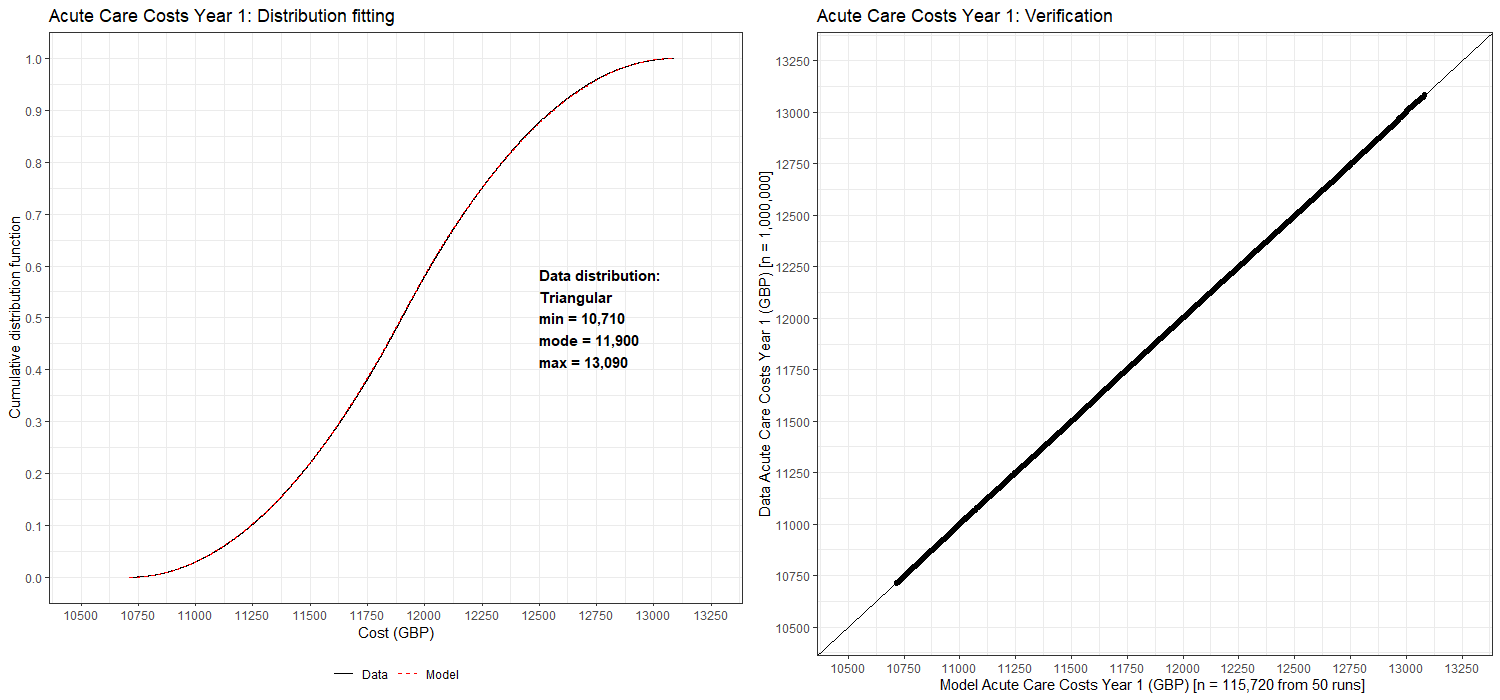


Figure 3: Acute Care Cost (Year 1) validation: Distribution fitting. Results from 50 simulation runs compared to large sample

## Years 2+

**(Input-3, B76:C82; Model results from 50 simulation runs, all years)**

The acute care costs for year 2+ at the time given by the data at the time and discussion with stakeholders was £2,430. To incorporate variability triangular distributions with a minimum and maximum of ± 10% of the mean, see Figure 4, for the treatment costs for all model generated patients, plotted against a bootstrapped sample of the distribution with the given parameters.

**
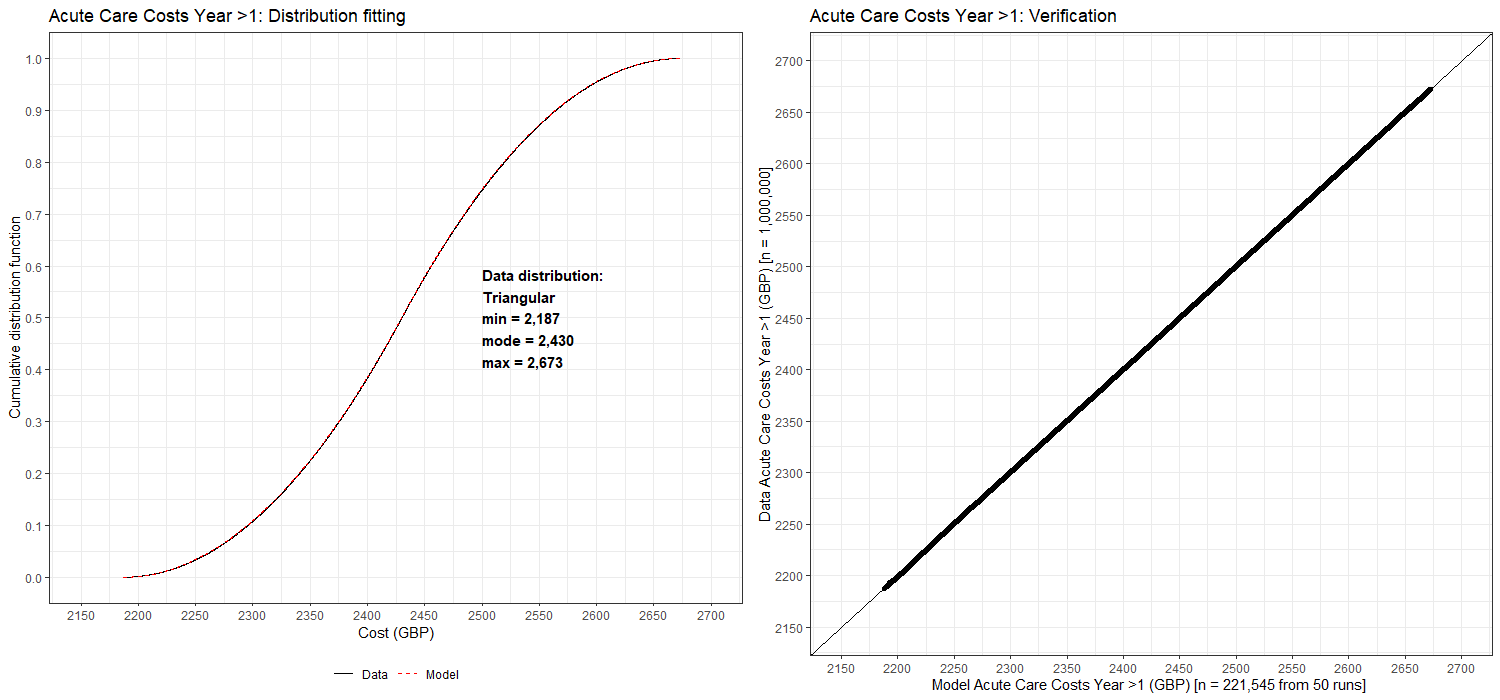
**

Figure 4: Acute Care Cost (Year 2+) validation: Distribution fitting. Results from 50 simulation runs compared to large sample

# Stroke Death Costs

**(Input-3, B76:C82; Model results from 50 simulation runs, all years)**

The direct costs associated with death at the time given by the data at the time and discussion with stakeholders was £400. To incorporate variability triangular distributions with a minimum and maximum of ± 10% of the mean, see Figure 5, for the treatment costs for all model generated patients, plotted against a bootstrapped sample of the distribution with the given parameters.

**
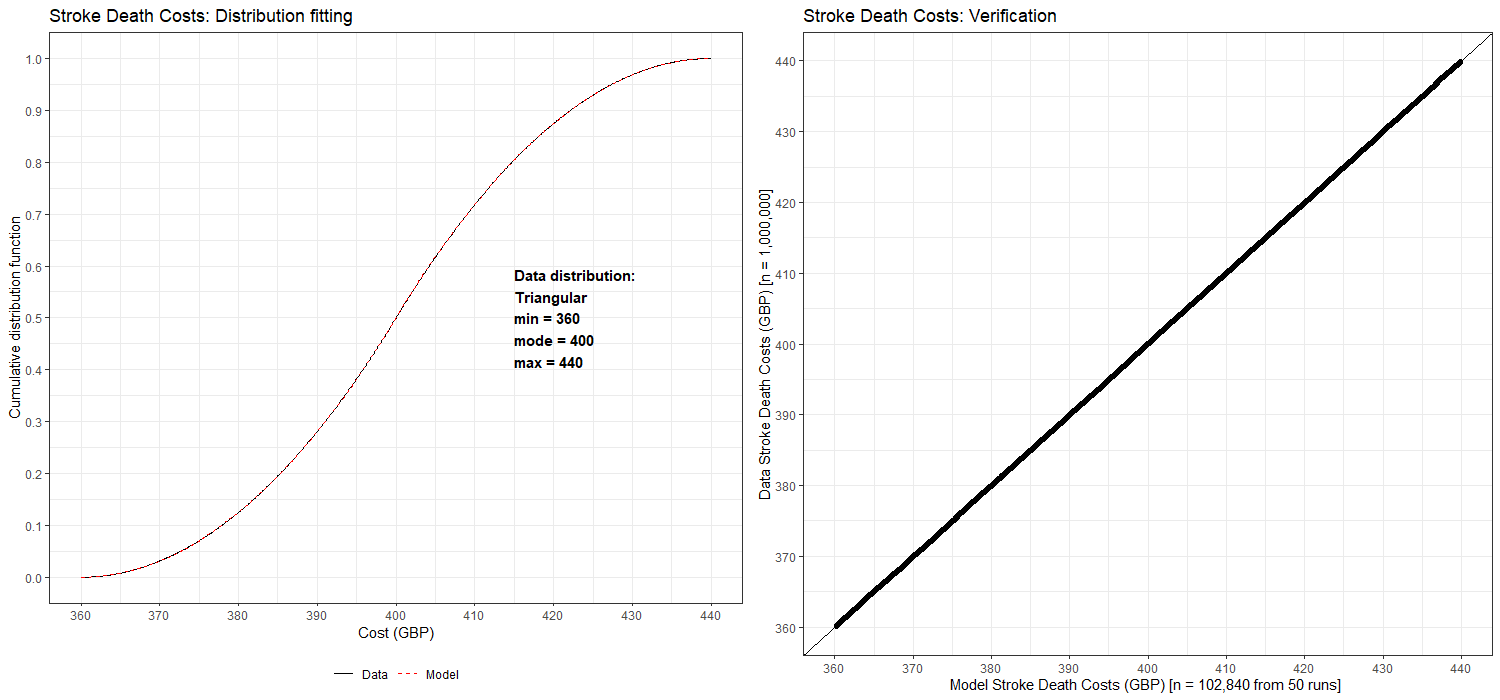
**

Figure 5: Stroke Death Costs validation: Distribution fitting. Results from 50 simulation runs compared to large sample

# CHADS2 parts

**(Input-2, H9:J16; Model results from 50 simulation runs, all years)**

Table 3 compares the model generated, CHADS2 constituent parts to the data in Appendix A-

Table 3: CHADS2 score constituent parts validation. Results from 50 simulation runs and 95% confidence intervals (CI).

| **Variable** | **Input**  **Absent** | **Model Absent** | **CI** | **Input**  **Present** | **Model Present** | **Model CI** |
| --- | --- | --- | --- | --- | --- | --- |
| Stroke | 74.76 | 73.43 | 73.31-73.54 | 25.24 | 26.57 | 26.46-26.69 |
| Hypertension | 43.23 | 43.16 | 43.01-43.30 | 56.77 | 56.85 | 56.70-56.99 |
| Diabetes | 79.16 | 79.17 | 79.06-79.29 | 20.84 | 20.83 | 20.71-20.94 |
| Congestion | 85.76 | 85.83 | 85.74-85.93 | 14.24 | 14.17 | 14.07-14.26 |

# Treatment Allocation

**(Input-3, B10:J16; Model results from 50 simulation runs, in year 1)**

During scenario experimentation the allocation of patients into treatment groups is a key input. Table 4 compares the model generated treatment allocations into the three treatment groups, 1) no treatment, 2) treatment with Warfarin and 3) treatment with NOAC.

Table 4: Treatment allocation based on CHADS2 score. Results from 50 simulation runs and 95% confidence intervals (CI). C = CHADS2 score

| **C** | **Input NoTreat** | **Model NoTreat** | **Model CI** | **Input Warfarin** | **Model Warfarin** | **Model CI** | **Input NOAC** | **Model NOAC** | **Model CI** |
| --- | --- | --- | --- | --- | --- | --- | --- | --- | --- |
| 0 | 57.00 | 56.91 | 56.49-57.33 | 20.30 | 20.41 | 19.96-20.85 | 22.70 | 22.69 | 22.23-23.14 |
| 1 | 31.32 | 31.34 | 31.12-31.57 | 38.24 | 38.06 | 37.85-38.27 | 30.44 | 30.60 | 30.32-30.88 |
| 2 | 25.10 | 25.01 | 24.82-25.20 | 42.52 | 42.68 | 42.39-42.97 | 32.38 | 32.32 | 32.06-32.56 |
| 3 | 23.05 | 23.03 | 22.71-23.36 | 43.58 | 43.69 | 43.41-43.96 | 33.37 | 33.28 | 32.99-33.58 |
| 4 | 19.86 | 19.76 | 19.41-20.11 | 42.88 | 42.91 | 42.44-43.38 | 37.25 | 37.33 | 36.89-37.78 |
| 5 | 22.53 | 22.58 | 21.79-23.38 | 40.00 | 40.14 | 39.33-40.96 | 37.47 | 37.27 | 36.38-38.17 |
| 6 | 23.94 | 24.45 | 21.94-26.96 | 40.85 | 39.76 | 37.17-42.35 | 35.21 | 35.79 | 32.86-38.72 |

# Warfarin Management

**(Input-3, L14:O17; Model results from 50 simulation runs, in year 1)**

For those patients allocated to the treatment with Warfarin group the model accurately replicated the compliance level of the patients, see Table 5.

Table 5: Warfarin Treatment Compliance level. Results from 50 simulation runs and 95% confidence intervals (CI). TTR = Time in Therapeutic Range

| **Good/poor compliance based on 65% TTR** | **Input**  **Percent (%)** | **Model**  **Percent (%)** | **Model CI** |
| --- | --- | --- | --- |
| Poor (<65%) | 22.07 | 22.02 | 21.82-22.22 |
| Good (≥65%) | 77.93 | 77.98 | 77.78-78.18 |

# Life Expectancy

**(Input-2, T8:V70; Model results from 50 simulation runs, all years)**

For those individuals who suffer a stroke and subsequently pass away, there expected life expectancy, years of lost life are calculated. Table 6, provides the expected life expectancy based on data available at the time, see Appendix A. The data is provided by gender and age. The “Age” column is the age of the person when they pass away. The “Input” column is the expected life expectancy. The model, sum and check columns are used to validate the expected life expectancy. The Check column is the sum column/count column. Please note that the Check column is rounded in the simulation model due to the annual time steps.

Table 6: Life expectancy for those who suffer a stroke. Results from 50 simulation runs and 95% confidence intervals (CI).

| **Age** | **Input Male** | **Model Count M** | **Sum Male** | **Check Male** | **Input Female** | **Model Count F** | **Sum Female** | **Check Female** |
| --- | --- | --- | --- | --- | --- | --- | --- | --- |
| 40 | 40.66 | 3 | 123 | 41 | 43.92 | 0 | 0 | 0 |
| 41 | 39.72 | 9 | 360 | 40 | 42.96 | 4 | 172 | 43 |
| 42 | 38.78 | 12 | 468 | 39 | 42 | 5 | 210 | 42 |
| 43 | 37.84 | 28 | 1064 | 38 | 41.04 | 6 | 246 | 41 |
| 44 | 36.91 | 27 | 999 | 37 | 40.09 | 8 | 320 | 40 |
| 45 | 35.98 | 40 | 1440 | 36 | 39.14 | 12 | 468 | 39 |
| 46 | 35.06 | 41 | 1435 | 35 | 38.19 | 20 | 760 | 38 |
| 47 | 34.13 | 66 | 2244 | 34 | 37.24 | 35 | 1295 | 37 |
| 48 | 33.22 | 71 | 2343 | 33 | 36.3 | 28 | 1008 | 36 |
| 49 | 32.3 | 104 | 3328 | 32 | 35.36 | 33 | 1155 | 35 |
| 50 | 31.39 | 122 | 3782 | 31 | 34.42 | 44 | 1496 | 34 |
| 51 | 30.49 | 130 | 3900 | 30 | 33.49 | 57 | 1881 | 33 |
| 52 | 29.59 | 152 | 4560 | 30 | 32.57 | 63 | 2079 | 33 |
| 53 | 28.69 | 206 | 5974 | 29 | 31.65 | 76 | 2432 | 32 |
| 54 | 27.81 | 167 | 4676 | 28 | 30.73 | 73 | 2263 | 31 |
| 55 | 26.92 | 219 | 5913 | 27 | 29.82 | 96 | 2880 | 30 |
| 56 | 26.05 | 270 | 7020 | 26 | 28.91 | 130 | 3770 | 29 |
| 57 | 25.18 | 280 | 7000 | 25 | 28.01 | 134 | 3752 | 28 |
| 58 | 24.33 | 350 | 8400 | 24 | 27.12 | 134 | 3618 | 27 |
| 59 | 23.48 | 345 | 7935 | 23 | 26.23 | 142 | 3692 | 26 |
| 60 | 22.64 | 413 | 9499 | 23 | 25.34 | 156 | 3900 | 25 |
| 61 | 21.82 | 426 | 9372 | 22 | 24.47 | 176 | 4224 | 24 |
| 62 | 21 | 497 | 10437 | 21 | 23.6 | 204 | 4896 | 24 |
| 63 | 20.19 | 549 | 10980 | 20 | 22.74 | 223 | 5129 | 23 |
| 64 | 19.4 | 623 | 11837 | 19 | 21.89 | 236 | 5192 | 22 |
| 65 | 18.61 | 696 | 13224 | 19 | 21.04 | 269 | 5649 | 21 |
| 66 | 17.83 | 791 | 14238 | 18 | 20.2 | 290 | 5800 | 20 |
| 67 | 17.05 | 897 | 15249 | 17 | 19.36 | 318 | 6042 | 19 |
| 68 | 16.29 | 913 | 14608 | 16 | 18.54 | 389 | 7391 | 19 |
| 69 | 15.53 | 1019 | 16304 | 16 | 17.73 | 420 | 7560 | 18 |
| 70 | 14.8 | 1151 | 17265 | 15 | 16.92 | 456 | 7752 | 17 |
| 71 | 14.07 | 1834 | 25676 | 14 | 16.13 | 756 | 12096 | 16 |
| 72 | 13.36 | 1746 | 22698 | 13 | 15.35 | 776 | 11640 | 15 |
| 73 | 12.68 | 1787 | 23231 | 13 | 14.59 | 740 | 11100 | 15 |
| 74 | 12 | 1792 | 21504 | 12 | 13.84 | 786 | 11004 | 14 |
| 75 | 11.35 | 2048 | 22528 | 11 | 13.11 | 907 | 11791 | 13 |
| 76 | 10.72 | 2303 | 25333 | 11 | 12.39 | 1040 | 12480 | 12 |
| 77 | 10.09 | 2466 | 24660 | 10 | 11.69 | 1076 | 12912 | 12 |
| 78 | 9.48 | 2657 | 23913 | 9 | 10.99 | 1159 | 12749 | 11 |
| 79 | 8.89 | 2729 | 24561 | 9 | 10.32 | 1294 | 12940 | 10 |
| 80 | 8.32 | 2873 | 22984 | 8 | 9.67 | 1350 | 13500 | 10 |
| 81 | 7.78 | 2905 | 23240 | 8 | 9.05 | 1319 | 11871 | 9 |
| 82 | 7.25 | 2922 | 20454 | 7 | 8.45 | 1371 | 10968 | 8 |
| 83 | 6.76 | 2888 | 20216 | 7 | 7.87 | 1401 | 11208 | 8 |
| 84 | 6.3 | 2929 | 17574 | 6 | 7.32 | 1436 | 10052 | 7 |
| 85 | 5.85 | 2944 | 17664 | 6 | 6.81 | 1395 | 9765 | 7 |
| 86 | 5.44 | 2737 | 13685 | 5 | 6.32 | 1339 | 8034 | 6 |
| 87 | 5.05 | 2717 | 13585 | 5 | 5.85 | 1306 | 7836 | 6 |
| 88 | 4.68 | 2556 | 12780 | 5 | 5.42 | 1252 | 6260 | 5 |
| 89 | 4.34 | 2541 | 10164 | 4 | 5.01 | 1150 | 5750 | 5 |
| 90 | 4.03 | 2360 | 9440 | 4 | 4.63 | 952 | 4760 | 5 |
| 91 | 3.73 | 2118 | 8472 | 4 | 4.29 | 846 | 3384 | 4 |
| 92 | 3.45 | 1813 | 5439 | 3 | 3.96 | 713 | 2852 | 4 |
| 93 | 3.21 | 1586 | 4758 | 3 | 3.66 | 602 | 2408 | 4 |
| 94 | 3.01 | 1385 | 4155 | 3 | 3.41 | 490 | 1470 | 3 |
| 95 | 2.8 | 1122 | 3366 | 3 | 3.17 | 399 | 1197 | 3 |
| 96 | 2.62 | 937 | 2811 | 3 | 2.94 | 299 | 897 | 3 |
| 97 | 2.42 | 810 | 1620 | 2 | 2.72 | 270 | 810 | 3 |
| 98 | 2.27 | 630 | 1260 | 2 | 2.53 | 186 | 558 | 3 |
| 99 | 2.14 | 520 | 1040 | 2 | 2.37 | 158 | 316 | 2 |
| 100 | 2.01 | 423 | 846 | 2 | 2.24 | 123 | 246 | 2 |

# Disability weight

**(Input-2, O9:Q13; Model results from 50 simulation runs, all years)**

The Disability weights as derived from the literature see Appendix A were valid for types of strokes considered in the model, see Table 7.

Table 7: Disability weight validation, from 50 simulation runs and 95% confidence intervals (CI)

| **Stroke type** | **Input**  **weight** | **Model**  **weight** | **Model**  **CI** |
| --- | --- | --- | --- |
| stroke | 0.019 | 0.019 | 0.019-0.019 |
| ischaemic | 0.070 | 0.070 | 0.070-0.070 |
| haemorrhagic | 0.552 | 0.552 | 0.552-0.552 |

# Mortality All-Cause

**(Input-3, B21:F33; Model results from 50 simulation runs, all years)**

Mortality in the model is broken into mortality related to Stroke and All-Cause mortality. The model All-Cause mortality resembles the model input data as depicted in Table 8.

Table 8: Mortality All-Cause by age band validation, from 50 simulation runs and 95% confidence intervals (CI).

| **Age band** | | **Input Female** | **Model Female** | **Model**  **CI** | **Input Male** | **Model Male** | **Model**  **CI** |
| --- | --- | --- | --- | --- | --- | --- | --- |
| 40 | 44 | 0.097 | 0.077 | 0.000-0.292 | 0.167 | 0.226 | 0.011-0.442 |
| 45 | 49 | 0.154 | 0.132 | 0.044-0.221 | 0.242 | 0.245 | 0.156-0.333 |
| 50 | 54 | 0.240 | 0.229 | 0.161-0.298 | 0.358 | 0.268 | 0.199-0.336 |
| 55 | 59 | 0.365 | 0.366 | 0.302-0.430 | 0.559 | 0.576 | 0.512-0.640 |
| 60 | 64 | 0.586 | 0.569 | 0.497-0.641 | 0.916 | 0.931 | 0.860-1.003 |
| 65 | 69 | 0.903 | 0.892 | 0.822-0.961 | 1.370 | 1.429 | 1.360-1.499 |
| 70 | 74 | 1.511 | 1.509 | 1.434-1.583 | 2.261 | 2.264 | 2.190-2.339 |
| 75 | 79 | 2.574 | 2.562 | 2.495-2.629 | 3.802 | 3.717 | 3.650-3.785 |
| 80 | 84 | 4.964 | 4.987 | 4.888-5.086 | 6.770 | 6.729 | 6.630-6.828 |
| 85 | 89 | 9.286 | 9.281 | 9.146-9.415 | 12.001 | 12.064 | 11.930-12.198 |
| 90 | 104 | 19.965 | 20.024 | 19.836-20.212 | 22.665 | 22.506 | 22.318-22.694 |

# Mortality Stroke

**(Input-3, H21:L24; Model results from 50 simulation runs, all years)**

The model Stroke mortality resembles the model input data as depicted in Table 9.

Table 9: Mortality Stroke by age band validation, from 50 simulation runs and 95% confidence intervals (CI).

| **Stroke** | **Measure** | **Female (%)** | **Male (%)** |
| --- | --- | --- | --- |
| >70 years old (%) | Input (%) | 36 | 32 |
|  | Model Mean (%) | 36.198 | 31.982 |
|  | Model CI (%) | 35.873-36.523 | 31.768-32.196 |
| ≤70 years old (%) | Input (%) | 27 | 19 |
|  | Model Mean (%) | 26.913 | 18.996 |
|  | Model CI (%) | 25.205-28.621 | 18.296-19.697 |
| Ischaemic (%) | Input (%) | 20 | 20 |
|  | Model Mean (%) | 19.731 | 20.214 |
|  | Model CI (%) | 18.968-20.493 | 19.816- 20.612 |
| Haemorrhagic (%) | Input (%) | 59 | 59 |
|  | Model Mean (%) | 63.572 | 58.994 |
|  | Model CI (%) | 60.586- 66.559 | 57.222- 60.768 |
