# Supplementary material for: Leveraging quality improvement initiatives to support development of decision support tools in healthcare
Source: Health Syst (Basingstoke). 2025 May 5;14(4):323–36. doi: 10.1080/20476965.2025.2500285 (PMC12777901; doi:10.1080/20476965.2025.2500285)
Supplement: Appendix C PRISMA literature review.docx [file THSS_A_2500285_SM4848.docx]

**Identification of new studies via backward reference searching**

**Identification of new studies via databases**

Records excluded:

Physical simulation (n = 5)

Not relevant (n = 5)

Medical (n = 17)

QI other (n = 5)

Support articles (n = 5)

Records excluded:

Pollution & environment (n = 24)

Physical simulation (n = 87)

Not relevant (n = 3)

Medical (n = 4)

Reports excluded:

Inaccessible (n = 2)

Duplicates (n = 2)

Accessible

(n = 55)

Titles screened

(n = 56)

Records identified from*:

Databases (n = 174)

Records identified though backward reference search:

Search (n = 59)

**Identification**

**Screening**

Titles and abstracts screened

(n = 31)

Abstracts screened

(n = 19)

Reports excluded:

Clinical only (n = 15)

Patient simulator (n = 1)

Clinical review (n = 1)

QI/implementation guidance (n = 2)

Simulation guidance (n = 2)

Patient & situational sim (n = 1)

Clinical, patient, & situational sim (n = 2)

Full-text articles assessed

(n = 17)

Reports excluded:

Not computer simulation (n = 4)

Sufficient coverage (n = 8)

Reports excluded:

Review (n = 1)

Accounting (n = 1)

Full-text articles assessed

(n = 19)

Total studies included

(n = 36)

* Web of Science search - Initial search 07.09.2021. Checked again 18.12.2023.

AB=(“quality improvement”) AND AB = (simulat*) AND AB = (health)

^a^Page, M. J., McKenzie, J. E., Bossuyt, P. M., Boutron, I., Hoffmann, T. C., Mulrow, C. D., et al. (2021). The PRISMA 2020 statement: an updated guideline for reporting systematic reviews. *BMJ (Online)*, *372*, n71–n71. <https://doi.org/10.1136/bmj.n71>

**Included**
