# Supplementary material for: Leveraging quality improvement initiatives to support development of decision support tools in healthcare
Source: Health Syst (Basingstoke). 2025 May 5;14(4):323–36. doi: 10.1080/20476965.2025.2500285 (PMC12777901; doi:10.1080/20476965.2025.2500285)
Supplement: Appendix B STRESS documentation.docx [file THSS_A_2500285_SM4847.docx]

**Strengthening the Reporting of Empirical Simulation Studies (STRESS)**

This table reports the models against the STRESS-DES guidelines, indicating where information is given in the paper or providing the details.

| Section/Subsection | Item | Model |
| --- | --- | --- |
| **1. Objectives** |  |  |
| Purpose of the model | 1.1 | See section 3.2. and section 3.3 |
| Model Outputs | 1.2 | Data recorded at an aggregate level after 1 year, 5 years, 10 years, and the end of the simulation, when all the patients in the model have passed away:   - Number of stroke, ischaemic stroke, and haemorrhagic stroke events - Number of stroke, ischaemic stroke, and haemorrhagic stroke deaths - Stroke, ischaemic stroke, and haemorrhagic stroke acute care cost - Stroke, ischaemic stroke, and haemorrhagic stroke treatment cost - Stroke, ischaemic stroke, and haemorrhagic stroke death cost - Disability-adjusted life year (DALY) |
| Experimental Aims | 1.3 | See section 4.3. |
| **2. Logic** |  |  |
| Base model overview diagram | 2.1 | See Figure 1 and flow chart below. |
| 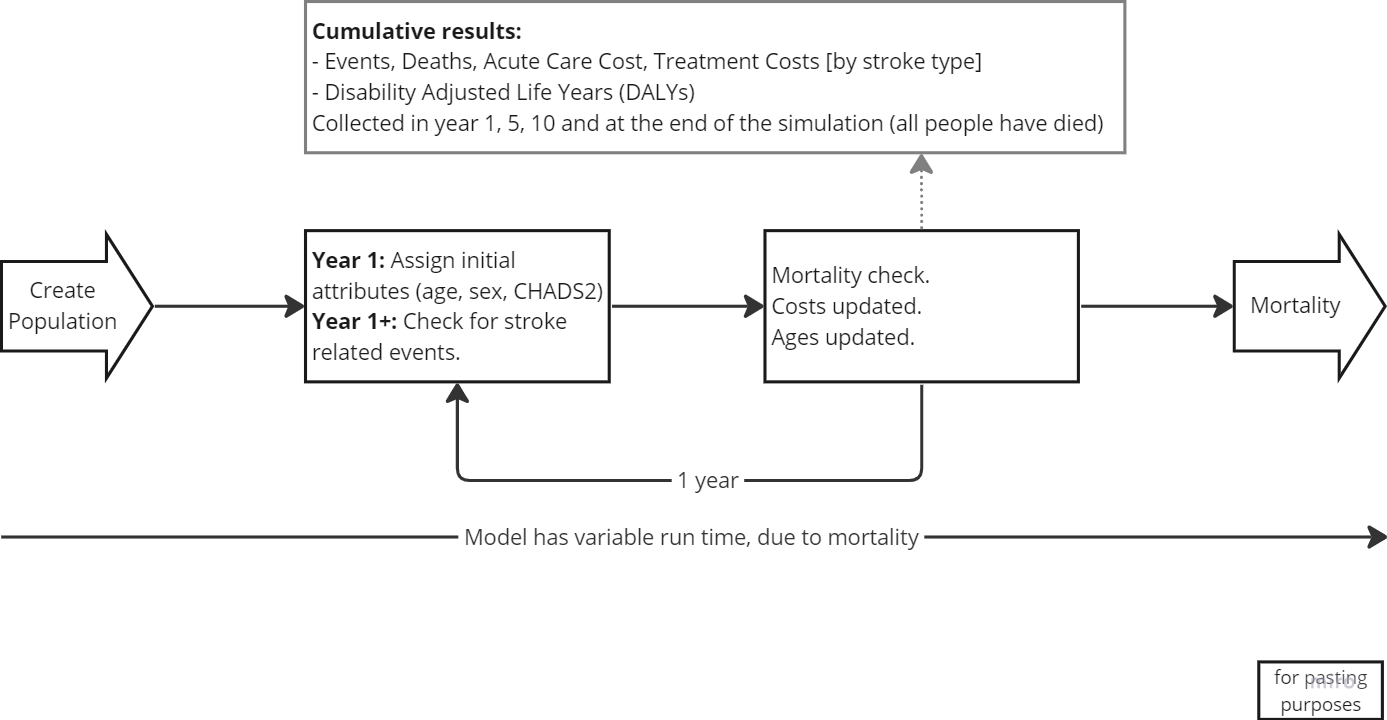 | | |
| Base model logic | 2.2 | See section 3.3 |
| Scenario logic | 2.3 | Scenarios are created by changing the input data fed to the Simul8 model via a Microsoft Excel User Interface.  See section 4.3. |
| Algorithms | 2.4 | See section 3.3. |
| Components | 2.5 | The model entities are people who move through the system the attributes recorded for them are:   - Person ID - Age - Gender - Life expectancy - Bleeding attribute if a bleed has occurred - Congestive history attribute - Hypertension history attribute - Vascular history attribute - CHADS2 score - Stroke history attribute - Cumulative number of strokes - Number of years since last stroke - Treatment (0 = no treatment, 1 = NOAC, 2 = Warfarin) - Annual stroke care cost if ever had stroke - Annual stroke treatment costs if ever had stroke - Mortality cost if ever had stroke - Compliance level if on Warfarin (good/bad)   The entire population is created at once and the model is run until everyone has died, due to stroke or reaching their life expectancy.  Stroke treatment regimens are defined and set when the population is created based on input data provided through the Excel User Interface, see section 3.4 and appendix A.  Patient attributes are sampled from empirical distributions see section 3.2 and appendix A. |
| **3. Data** |  |  |
| Data sources | 3.1 | - See section 3.2 and appendix A. - Data from Quality Improvement Exercise, Don’t wait to anticoagulate (DWAC). The data were delivered in two spreadsheets; the “Before” and “After” the implementation of the intervention. The “Before” has 9,582 records/patients and the “After” has 10,300. Most of the patients between the two spreadsheets appear to be the same with some missing data due to death, relocating outside the catchment area or new patients having registered with GPs. For the purposes of this project and the modelling study, only the “After” data was used as a before-after comparison was outside the scope of this project. - Literature for clinical trajectories and costs. |
| Pre-processing | 3.2 | Empirical distributions are used |
| Input parameters | 3.3 | Empirical distributions are used. |
| Assumptions | 3.4 | - The stroke treatment regimens and other population attributes are sampled from empirical distributions when the population is created. - Stroke and mortality probabilities are determined annually, and other attributes adjusted accordingly. - Treatment, care, and mortality costs values assumed to be constant throughout the model time horizon (~55-70 years), with a Triangular Distribution of ±10% of the input value. - Multiple strokes do not result in increased mortality or DALY weights. - Season variations are not considered. |
| **4. Experimentation** |  |  |
| Initialisation | 4.1 | The simulation always begins with a population of 10,000 people.  The simulation is terminating. |
| Run length | 4.2 | The simulation runs until the 10,000 people have passed away. Results are collected after 1 year, 5 years, 10 years and at the end of the simulation |
| Estimation approach | 4.3 | 50 independent replications were used. See Figure 4 in section 4.1. |
| **5. Implementation** |  |  |
| Software or programming language | 5.1 | Simul8 2022 Professional Build 4367 |
| Random sampling | 5.2 | Commercial software was used see 5.1. |
| Model execution | 5.3 | Commercial software was used see 5.1. |
| System Specification | 5.4 | Simul8 2022 Professional Build 4367.  Run on a Lenova ThinkPad T14s with an 11th Gen Intel(R) Core(TM) i5-1135G7 @ 2.40GHz, 16.0 GB of RAM and a 64-bit operating system |
| **6. Code Access** |  |  |
| Computer Model Sharing Statement | 6.1 | The model is available upon reasonable request from the corresponding author. |
