# Supplementary material for: Leveraging quality improvement initiatives to support development of decision support tools in healthcare
Source: Health Syst (Basingstoke). 2025 May 5;14(4):323–36. doi: 10.1080/20476965.2025.2500285 (PMC12777901; doi:10.1080/20476965.2025.2500285)
Supplement: Appendix A data.docx [file THSS_A_2500285_SM4846.docx]

# User Interface, Input Data and Sensitivity Analysis

This appendix provides an overview of the User Interface, as with the simulation model the User Interface is available upon reasonable request from the corresponding author. Important input model data are presented, these data are entered into the User Interface and then imported into the simulation model. Selective sensitivity analyses of key model inputs are discussed.

## User Interface

Figure A-1 provides a sample of one of the User Interfaces model input sheets, part of the Input-2 sheet. The User Interface consists of three input sheets: Input-1, Input-2, and Input-3. **Input-1**, allows the user to specify the number of simulation runs in a model Trial, how model input data is read into the simulation model, the number of patients to be modelled, the random number seeds. **Input-2**, user specifies clinical and patient characteristics. Input-3, user specifies treatment probabilities, mortality rates, treatment management and costs.

Figure A‑1: Example screenshot from the Microsoft Excel User Interface

Figure A-2 presents a section of the aggregate model results which are collected in Year 1, Year 5, Year 10, and at the End of the simulation Run. Note that the model has no fixed end time as it is run until all the population has died. The three output sheets are **Output-3** (the output at the End of the Simulation), **Output-3_2** (the output at the end of year 1), **Output-3_6** (the output at the end of year 5), and **Output-3_11** (the output at the end of year 10).

Individual run results are collected and summary statistics (mean, standard deviation and 95% mean confidence intervals) are presented for the Events, Deaths, Acute Care Cost, Treatment Cost and Death Costs for three types of stroke:

1. Strokes, Other strokes e.g. not Ischaemic or Haemorrhagic and transient ischaemic attacks (TIAs)
2. Ischaemic strokes
3. Haemorrhagic strokes

In addition to the stroke related results disability adjusted life years (DALY) are collected.

Figure A‑2: Example screenshot from the Microsoft User Interface. Patient and System outcomes for the baseline scenario

## Input Model Data

The Health Innovation Network is the innovation arm of the NHS in England and is comprised of 15 regional networks across the country. Each regional network covers a number of diverse partners (providers of NHS care working with universities, industry, NHS commissioners and a wide range of other organisations) committed to equality and excellence, which will accelerate the spread of innovative, evidence-based practice to improve health and care quality.

The quality improvement initiative that is the topic of this study aimed at reducing the risk of stroke in people with Atrial Fibrillation (AF) and support an increase in the uptake of anticoagulation including new technology (NOACs) across the region in line with NICE evidence. It also aimed at optimising the use of anticoagulation and to see a decrease in the use of aspirin for stroke prevention in primary care. The QI project was a complex programme of interventions set within primary care and was implemented in different phases each with a specific target group at a different location.

Most data used in the development of the simulation model and tool were received from Phase 2 of the QI project. The data was rich in respect to the epidemiological aspects of the project which proved useful in describing the input population for our models. However, given that the dataset was not longitudinal in nature, it lacked several data items that were needed by the simulation model.

### Input parameters in the QI project data

Data from the QI project was used to define the size and characteristics of the population cohort that is used in the simulations. Anonymised data were received for 10,300 individuals in total. The variables in the data included:

- Age (Table A-1, Figure A-3)
- Gender (Figure A-3)
- Treatment type (Table A-2)
- Warfarin management (Figure A-4)
- CHA₂DS constituents (Table A-3)

Table A‑1: Gender distribution in the QI project patient population

| **Gender** | **Frequency** | **Percentage (%)** |
| --- | --- | --- |
| Male | 5,941 | 57,68 |
| Female | 4,359 | 42.32 |
| Total | 10,300 | 100.00 |


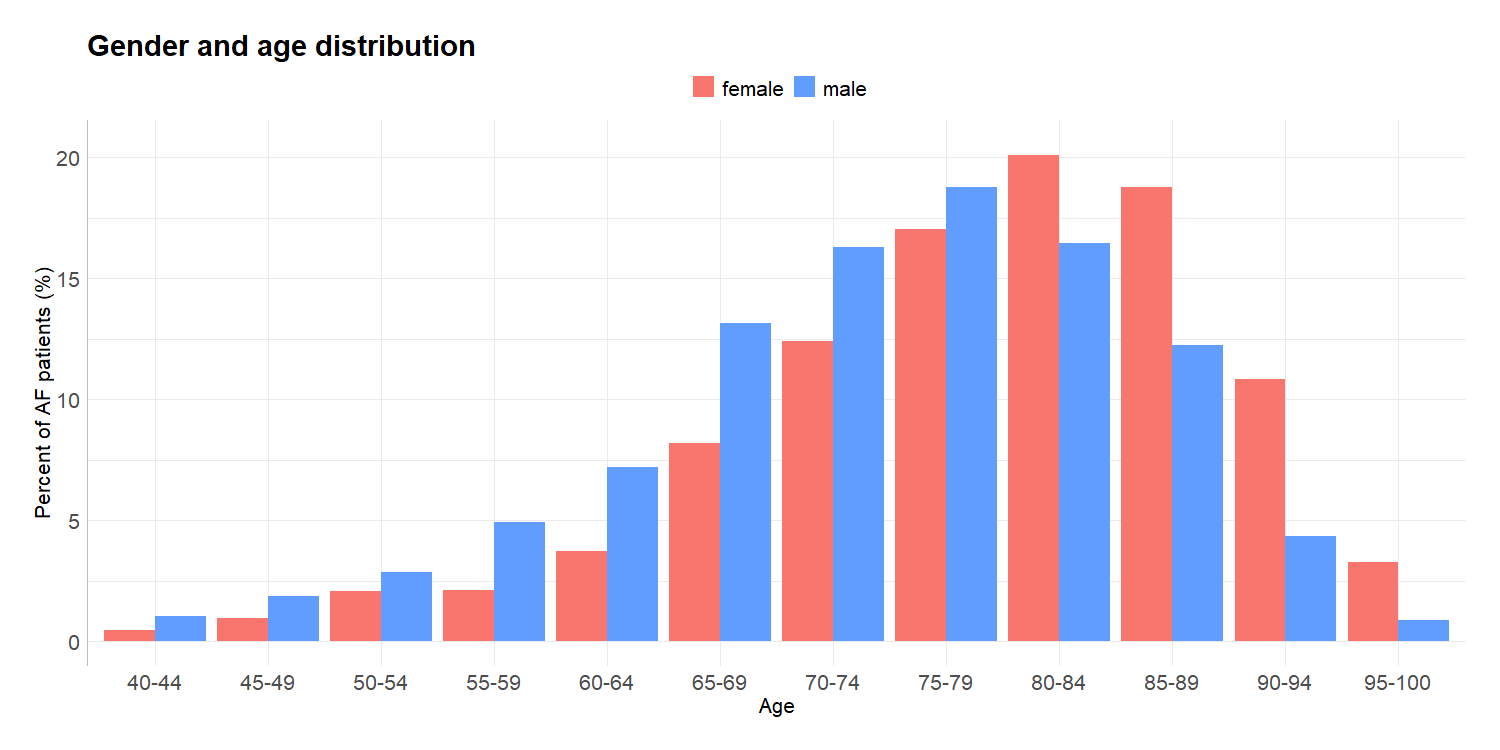


Figure A‑3: Gender and age distribution in the patient population

Table A‑2: Treatment proportions based on CHADS2 score in the QI project population data

| **CHADS_2_** | **No Treatment (%)** | **Warfarin (%)** | **NOAC (%)** |
| --- | --- | --- | --- |
| 0 | 57 % | 20 % | 23 % |
| 1 | 31 % | 38 % | 30 % |
| 2 | 25 % | 43 % | 32 % |
| 3 | 23 % | 44 % | 33 % |
| 4 | 20 % | 43 % | 37 % |
| 5 | 23 % | 40 % | 37 % |
| 6 | 24 % | 41 % | 35 % |
| **Total** | **30 %** | **38 %** | **31 %** |


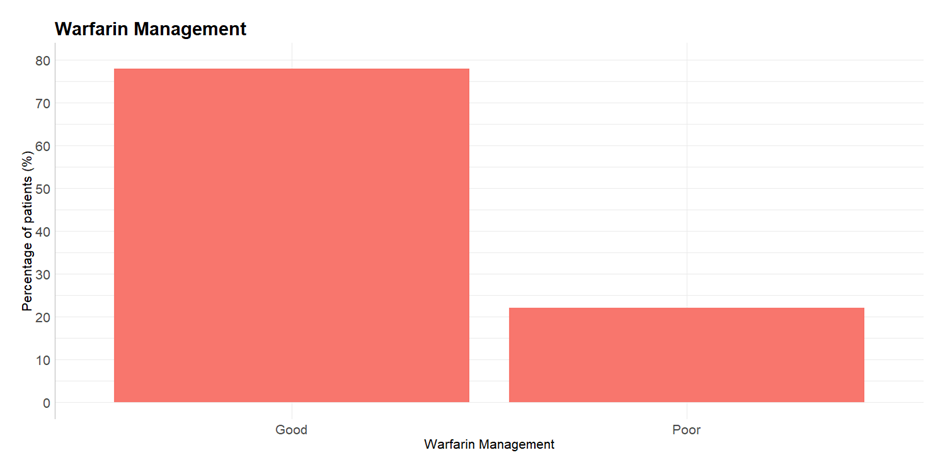


Figure A‑4: Warfarin management based on QI project population data

Table A‑3: CHADS_2_ score constituents

|  | **CHADS_2_ Distribution (%)** | |
| --- | --- | --- |
| **Variable** | **Absent** | **Present** |
| Age ≥75 | 39.96 | 60.04 |
| Stroke | 74.76 | 25.24 |
| Hypertension | 43.23 | 56.77 |
| Diabetes | 79.16 | 20.84 |
| Congestive heart failure | 85.76 | 14.24 |

## Input parameters from the literature

Sources of input data derived from the literature are presented in Table A-3.

Table A‑4: Sources of input data from the literature

| All-cause Mortality | | ONS 2015 (calculations) |
| --- | --- | --- |
| Stroke Mortality | Overall | Lee, S., Shafe, A. C., & Cowie, M. R. (2011). UK stroke incidence, mortality and cardiovascular risk management 1999-2008: time-trend analysis from the General Practice Research Database. *BMJ Open*, *1*(2), e000269. <https://doi.org/10.1136/bmjopen-2011-000269> |
|  | Ischaemic | Feigin, V. L., Lawes, C. M., Bennett, D. A., & Anderson, C. S. (2003). Stroke epidemiology: a review of population-based studies of incidence, prevalence, and case-fatality in the late 20th century. *Lancet Neurol*, *2*(1), 43-53. <https://doi.org/10.1016/s1474-4422(03)00266-7>  Grysiewicz, R. A., Thomas, K., & Pandey, D. K. (2008). Epidemiology of ischemic and hemorrhagic stroke: incidence, prevalence, mortality, and risk factors. *Neurol Clin*, *26*(4), 871-895, vii. <https://doi.org/10.1016/j.ncl.2008.07.003> |
|  | Haemorrhagic | Qureshi, A. I., Mendelow, A. D., & Hanley, D. F. (2009). Intracerebral haemorrhage. *LANCET*, *373*(9675), 1632-1644. <https://doi.org/10.1016/S0140-6736(09)60371-8>  Fogelholm, R., Murros, K., Rissanen, A., & Avikainen, S. (2005). Long term survival after primary intracerebral haemorrhage: a retrospective population based study. *J Neurol Neurosurg Psychiatry*, *76*(11), 1534-1538. <https://doi.org/10.1136/jnnp.2004.055145>  Flaherty, M. L., Haverbusch, M., Sekar, P., Kissela, B., Kleindorfer, D., Moomaw, C. J., Sauerbeck, L., Schneider, A., Broderick, J. P., & Woo, D. (2006). Long-term mortality after intracerebral hemorrhage. *Neurology*, *66*(8), 1182-1186. <https://doi.org/10.1212/01.wnl.0000208400.08722.7c> |
| Risk CHAD Stroke | | Gage, B. F., Waterman, A. D., Shannon, W., Boechler, M., Rich, M. W., & Radford, M. J. (2001). Validation of clinical classification schemes for predicting stroke: results from the National Registry of Atrial Fibrillation. *JAMA*, *285*(22), 2864-2870. <https://doi.org/10.1001/jama.285.22.2864> |
| Risk Ischaemic Stroke | | Ruff, C. T., Giugliano, R. P., Braunwald, E., Hoffman, E. B., Deenadayalu, N., Ezekowitz, M. D., Camm, A. J., Weitz, J. I., Lewis, B. S., Parkhomenko, A., Yamashita, T., & Antman, E. M. (2014). Comparison of the efficacy and safety of new oral anticoagulants with warfarin in patients with atrial fibrillation: a meta-analysis of randomised trials. *LANCET*, *383*(9921), 955-962. <https://doi.org/10.1016/S0140-6736(13)62343-0>  Calculations for pooling together |
| Risk Haemorrhagic Stroke | | Ruff, C. T., Giugliano, R. P., Braunwald, E., Hoffman, E. B., Deenadayalu, N., Ezekowitz, M. D., Camm, A. J., Weitz, J. I., Lewis, B. S., Parkhomenko, A., Yamashita, T., & Antman, E. M. (2014). Comparison of the efficacy and safety of new oral anticoagulants with warfarin in patients with atrial fibrillation: a meta-analysis of randomised trials. *LANCET*, *383*(9921), 955-962. <https://doi.org/10.1016/S0140-6736(13)62343-0>  Calculations for pooling together |
| Treatment Cost | NOAC | Regional Drug & Therapeutics Centre (RDTC). (2017). *Cost Comparison Charts* <https://rdtc.nhs.uk/prescribing-support-document/cost-comparison-charts/> |
|  | Warfarin | NHS England. (2017). *The GRASP-AF quality improvement tool has been developed by PRIMIS and delivered in partnership with NHS England* <https://www.england.nhs.uk/london/wp-content/uploads/sites/8/2019/08/1.2.25-Grasp-AF-Instruction-Booklet.pdf> |
| Strok Associated Costs | Stroke year 1 | NHS Improvement. (2009). *Commissioning for Stroke Prevention in Primary Care - The Role of Atrial Fibrillation* [https://webarchive.nationalarchives.gov.uk/ukgwa/20130513165155](https://webarchive.nationalarchives.gov.uk/ukgwa/20130513165155/http:/system.improvement.nhs.uk/ImprovementSystem/ViewDocument.aspx?path=Cardiac/National/Website/AF_Commissioning_Guide.pdf)  [/http://system.improvement.nhs.uk/ImprovementSystem/ViewDocument.aspx?path=Cardiac/](https://webarchive.nationalarchives.gov.uk/ukgwa/20130513165155/http:/system.improvement.nhs.uk/ImprovementSystem/ViewDocument.aspx?path=Cardiac/National/Website/AF_Commissioning_Guide.pdf)  [National/Website/AF_Commissioning_Guide.pdf](https://webarchive.nationalarchives.gov.uk/ukgwa/20130513165155/http:/system.improvement.nhs.uk/ImprovementSystem/ViewDocument.aspx?path=Cardiac/National/Website/AF_Commissioning_Guide.pdf)  Saka, O., McGuire, A., & Wolfe, C. (2009). Cost of stroke in the United Kingdom. *Age Ageing*, *38*(1), 27-32. <https://doi.org/10.1093/ageing/afn281>  Bayer Plc. (2011). *Submission to National Institute for Health and Clinical Excellence. Single Technology Appraisal (STA) of Rivaroxaban* <https://www.nice.org.uk/guidance/ta256/documents/atrial-fibrillation-stroke-prevention-rivaroxaban-bayer4> |
|  | Years 2-5 | NICE National Institute for Health and Care Excellence. (2012). *Dabigatran etexilate for the prevention of stroke and systemic embolism in atrial fibrillation [TA249]* <https://www.nice.org.uk/guidance/ta249>  NICE National Institute for Health and Care Excellence. (2012). *Rivaroxaban for the prevention of stroke and systemic embolism in people with atrial fibrillation [TA256]* <https://www.nice.org.uk/guidance/ta256> |
|  | Fatal Stroke | Bayer Plc. (2011). *Submission to National Institute for Health and Clinical Excellence. Single Technology Appraisal (STA) of Rivaroxaban* <https://www.nice.org.uk/guidance/ta256/documents/atrial-fibrillation-stroke-prevention-rivaroxaban-bayer4> |
| Disability weights | | Salomon, J. A., Haagsma, J. A., Davis, A., de Noordhout, C. M., Polinder, S., Havelaar, A. H., Cassini, A., Devleesschauwer, B., Kretzschmar, M., Speybroeck, N., Murray, C. J., & Vos, T. (2015). Disability weights for the Global Burden of Disease 2013 study. *Lancet Glob Health*, *3*(11), e712-723. <https://doi.org/10.1016/S2214-109X(15)00069-8> |

As many of the input parameter values could not be derived from the data provided, essential values such as mortality rates were obtained from the literature. After extensive search of the literature, we established input parameter values for:

- Stoke risk (Table A-4, Figure A-5, Figure A-6)
- All-cause mortality excluding stroke by age and gender (Figure A-7)
- Stoke mortality (Table A-5)
- Costs (Table A-6, Table A-7)
- DALYs (Table A-8)

Table A‑5: CHADS_2_ Score and probability of stroke per year[1, 2]

| **CHADS_2_ Score** | |
| --- | --- |
| **Score** | **Annual Stroke Risk** |
| 0 | 1.9 |
| 1 | 2.8 |
| 2 | 4 |
| 3 | 5.9 |
| 4 | 8.5 |
| 5 | 12.5 |
| 6 | 18.2 |


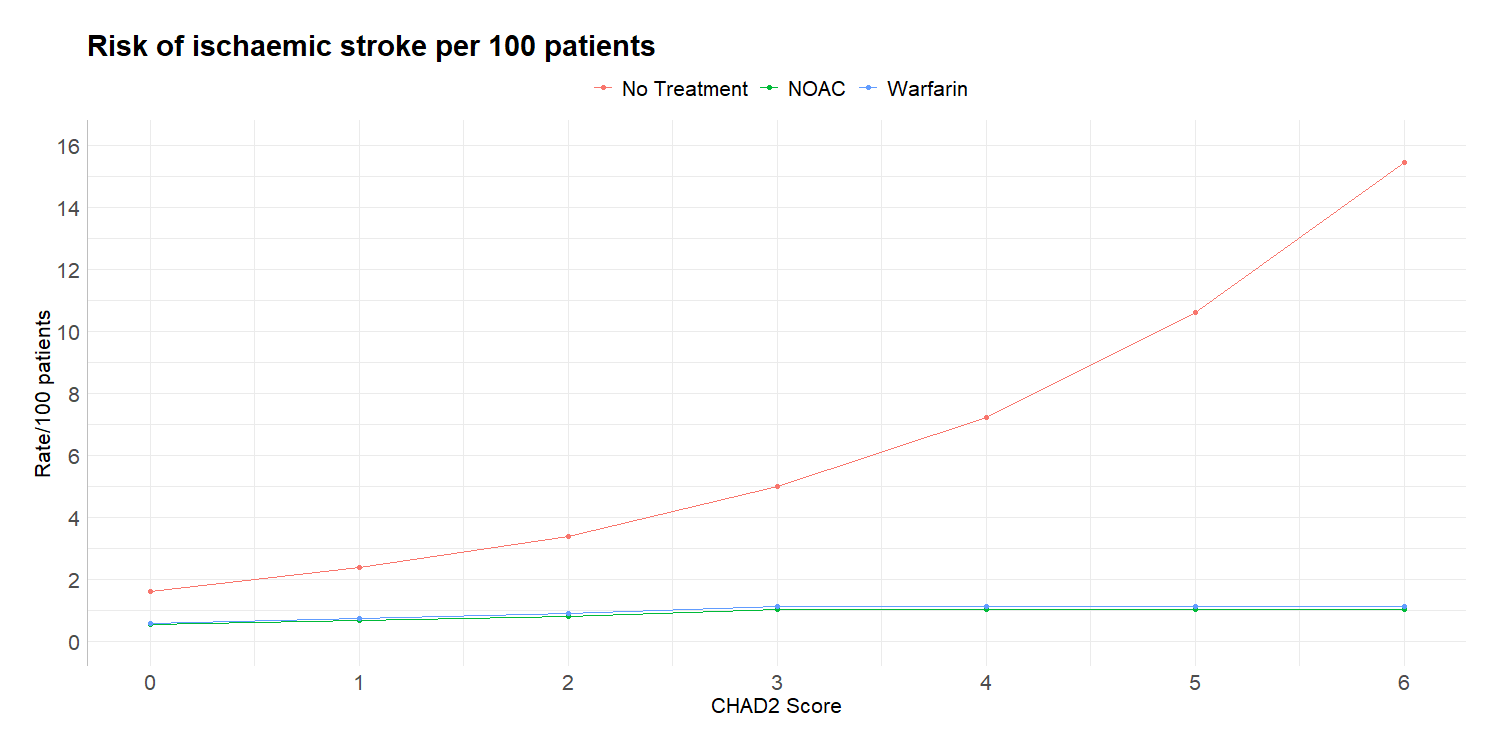


Figure A‑5: Risk of ischaemic stroke as found in literature and after data manipulation [3]


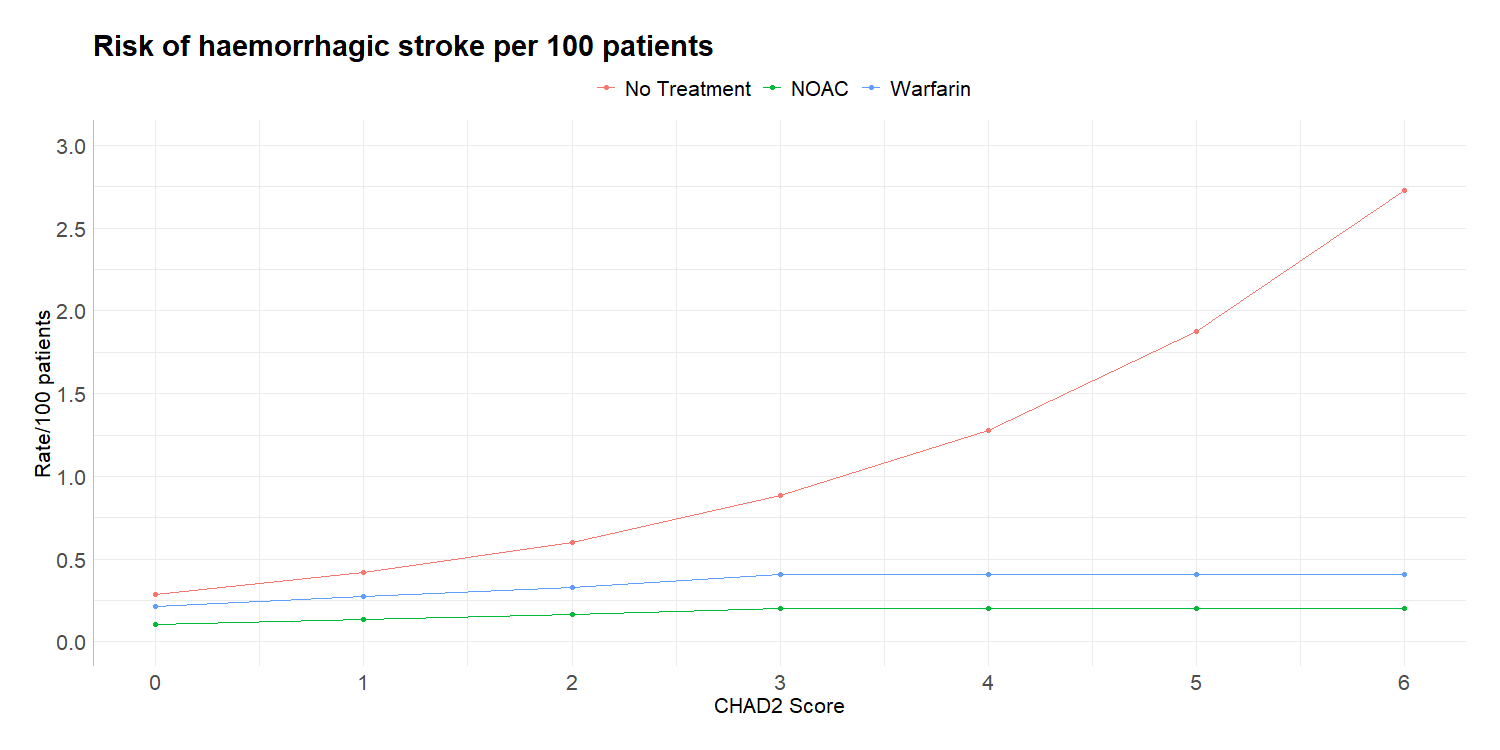


Figure A‑6: Risk of haemorrhagic stroke as found in literature and after data manipulation [3]


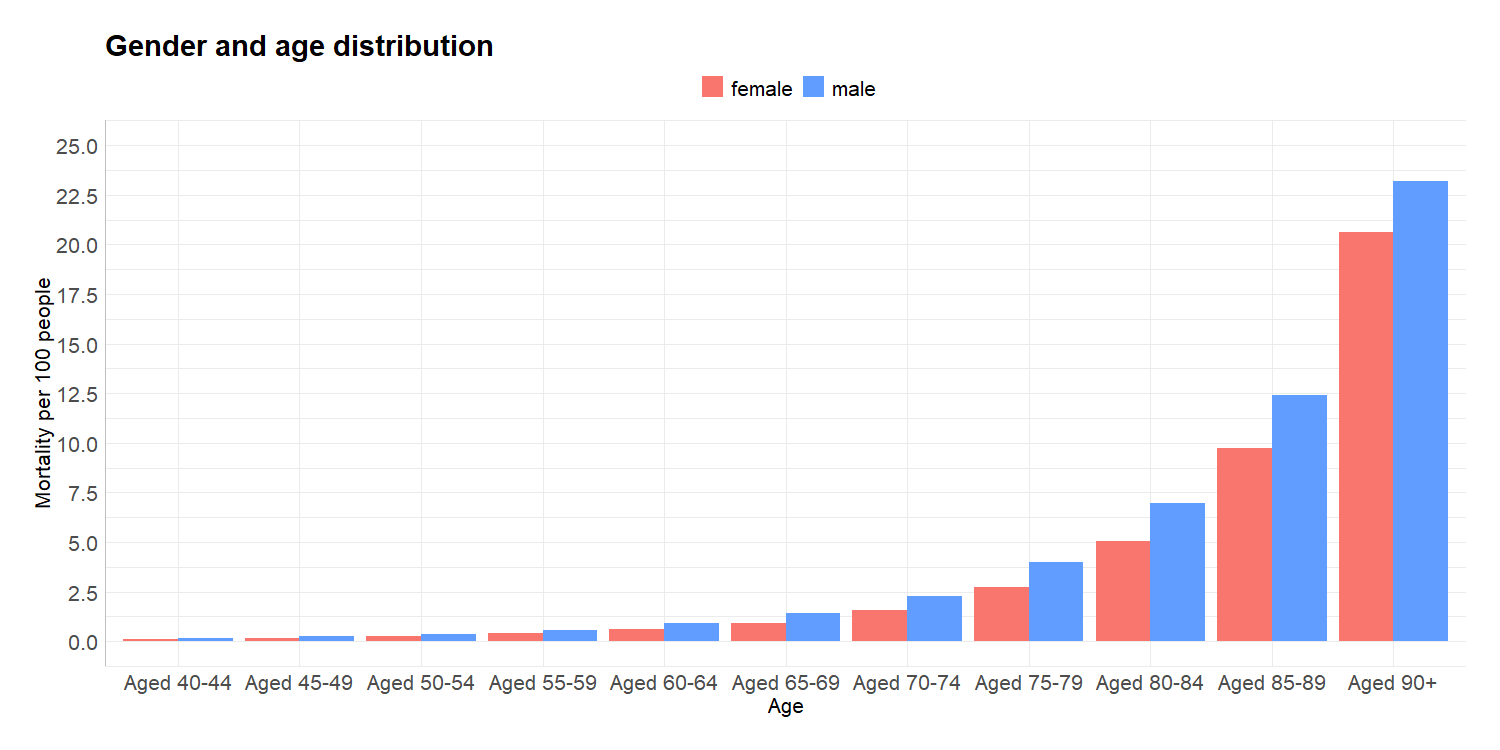


Figure A‑7: All-cause mortality by gender and age [4]

Table A‑6: Mortality due to stroke as found in the literature [5-7]

|  | **Mortality** | | | |
| --- | --- | --- | --- | --- |
| **Gender** | **>70 years old (%)** | **Overall (%)** | **Ischaemic (%)** | **Haemorrhagic (%)** |
| Male | 32 | 19 | 20 | 59 |
| Female | 36 | 27 | 20 | 59 |

Table A‑7: Costs of AF treatment based on the literature [8]

| **AF treatment** | **Cost per year (GBP)** |
| --- | --- |
| NOAC | 670 |
| Warfarin | 380 |

Table A‑8: Acute care costs of stroke based on the literature [9, 10]

| **Stroke status** | **Costs (GBP) per stroke** | **Min** | **Max** |
| --- | --- | --- | --- |
| Death | 400 | 360 | 440 |
| 1^st^ year | 11,900 | 10,000 | 13,800 |
| 2^nd^ year | 2,430 | 2,187 | 2,673 |
| 3^rd^ year | 2,430 | 2,187 | 2,673 |
| 4^th^ year | 2,430 | 2,187 | 2,673 |
| 5^th^ year | 2,430 | 2,187 | 2,673 |

Table A‑9: Disability weights for DALY calculation based on the literature [11]

| **Condition** | **Disability weight** |
| --- | --- |
| Long term consequence, Stroke | 0.019 |
| Long term consequence, Ischaemic | 0.07 |
| Long term consequence, Haemorrhagic | 0.552 |
| Death | 1 |

## Selective Sensitivity Analysis


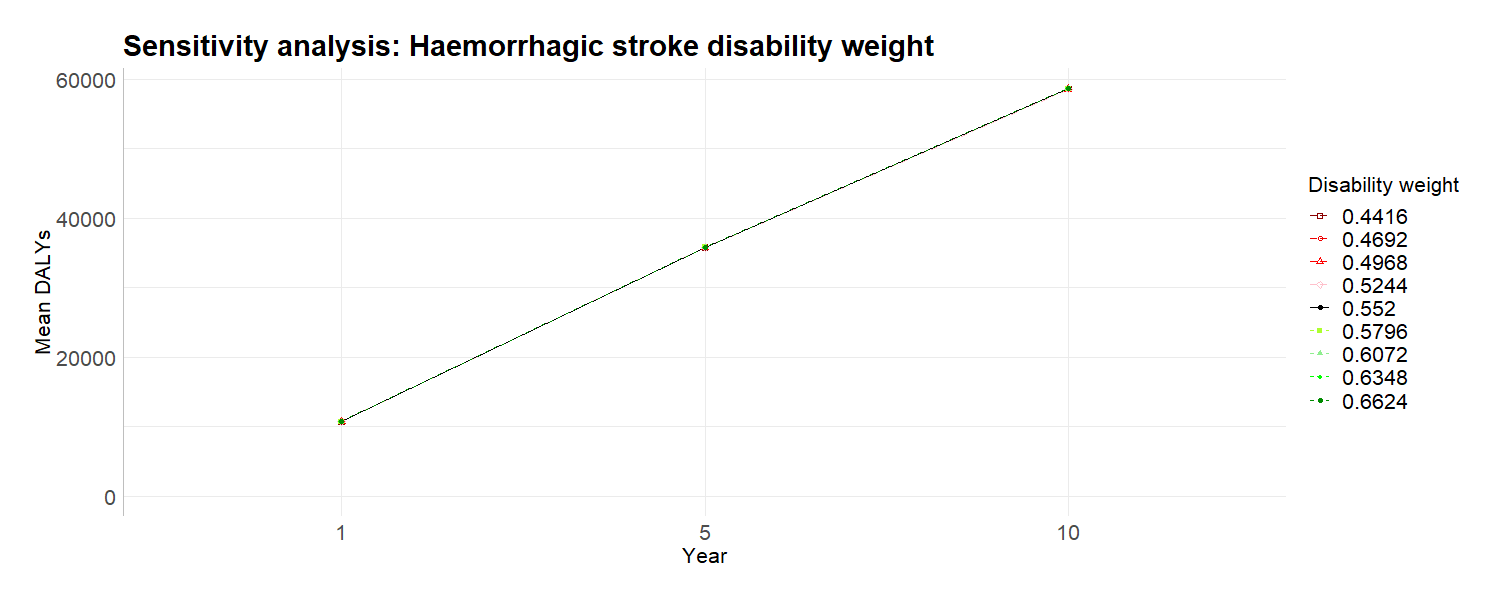


## References

1. Gage, B.F., et al., *Selecting patients with atrial fibrillation for anticoagulation: stroke risk stratification in patients taking aspirin.* Circulation, 2004. **110**(16): p. 2287-92.

2. Gage, B.F., et al., *Validation of clinical classification schemes for predicting stroke: results from the National Registry of Atrial Fibrillation.* JAMA, 2001. **285**(22): p. 2864-70.

3. Ruff, C.T., et al., *Comparison of the efficacy and safety of new oral anticoagulants with warfarin in patients with atrial fibrillation: a meta-analysis of randomised trials.* Lancet, 2014. **383**(9921): p. 955-62.

4. Office for National Statistics (ONS). *Deaths*. 2016 [accessed 25.02.2018]; Available from: <https://www.ons.gov.uk/peoplepopulationandcommunity/birthsdeathsandmarriages/deaths>.

5. Feigin, V.L., et al., *Stroke epidemiology: a review of population-based studies of incidence, prevalence, and case-fatality in the late 20th century.* Lancet Neurol, 2003. **2**(1): p. 43-53.

6. Lee, S., A.C. Shafe, and M.R. Cowie, *UK stroke incidence, mortality and cardiovascular risk management 1999-2008: time-trend analysis from the General Practice Research Database.* BMJ Open, 2011. **1**(2): p. e000269.

7. Qureshi, A.I., A.D. Mendelow, and D.F. Hanley, *Intracerebral haemorrhage.* Lancet, 2009. **373**(9675): p. 1632-44.

8. Regional Drug & Therapeutics Centre (RDTC). *Cost Comparison Charts*. 2015 [cited 2015; Available from: <https://rdtc.nhs.uk/prescribing-support-document/cost-comparison-charts/>.

9. National Centre for Health and Care Excellence (NICE). *Atrial fibrillation: diagnosis and management NICE guideline [NG196]*. 2021; Available from <https://www.nice.org.uk/guidance/ng196>

10. NHS Improvement. *Commissioning for Stroke Prevention in Primary Care - The Role of Atrial Fibrillation*. 2009; Available from: <https://webarchive.nationalarchives.gov.uk/ukgwa/20130513165155/http://system.improvement.nhs.uk/ImprovementSystem/ViewDocument.aspx?path=Cardiac/National/Website/AF_Commissioning_Guide.pdf>.

11. Salomon, J.A., et al., *Disability weights for the Global Burden of Disease 2013 study.* Lancet Glob Health, 2015. **3**(11): p. e712-23.
